# RNA-sequencing across three matched tissues reveals shared and tissue-specific gene expression and pathway signatures of COPD

**DOI:** 10.1186/s12931-019-1032-z

**Published:** 2019-04-02

**Authors:** Jarrett D. Morrow, Robert P. Chase, Margaret M. Parker, Kimberly Glass, Minseok Seo, Miguel Divo, Caroline A. Owen, Peter Castaldi, Dawn L. DeMeo, Edwin K. Silverman, Craig P. Hersh

**Affiliations:** 1 Channing Division of Network Medicine, Brigham and Women’s Hospital, 181 Longwood Avenue, Boston, MA 02115 USA; 2 Division of Pulmonary and Critical Care Medicine, Brigham and Women’s Hospital, Boston, MA 02115 USA

**Keywords:** COPD, Emphysema, Genomics, RNA-seq, Transcriptomics

## Abstract

**Background:**

Multiple gene expression studies have been performed separately in peripheral blood, lung, and airway tissues to study COPD. We performed RNA-sequencing gene expression profiling of large-airway epithelium, alveolar macrophage and peripheral blood samples from the same subset of COPD cases and controls from the COPDGene study who underwent bronchoscopy at a single center. Using statistical and gene set enrichment approaches, we sought to improve the understanding of COPD by studying gene sets and pathways across these tissues, beyond the individual genomic determinants.

**Methods:**

We performed differential expression analysis using RNA-seq data obtained from 63 samples from 21 COPD cases and controls (includes four non-smokers) via the R package DESeq2. We tested associations between gene expression and variables related to lung function, smoking history, and CT scan measures of emphysema and airway disease. We examined the correlation of differential gene expression across the tissues and phenotypes, hypothesizing that this would reveal preserved and private gene expression signatures. We performed gene set enrichment analyses using curated databases and findings from prior COPD studies to provide biological and disease relevance.

**Results:**

The known smoking-related genes *CYP1B1* and *AHRR* were among the top differential expression results for smoking status in the large-airway epithelium data. We observed a significant overlap of genes primarily across large-airway and macrophage results for smoking and airway disease phenotypes. We did not observe specific genes differentially expressed in all three tissues for any of the phenotypes. However, we did observe hemostasis and immune signaling pathways in the overlaps across all three tissues for emphysema, and amyloid and telomere-related pathways for smoking. In peripheral blood, the emphysema results were enriched for B cell related genes previously identified in lung tissue studies.

**Conclusions:**

Our integrative analyses across COPD-relevant tissues and prior studies revealed shared and tissue-specific disease biology. These replicated and novel findings in the airway and peripheral blood have highlighted candidate genes and pathways for COPD pathogenesis.

**Electronic supplementary material:**

The online version of this article (10.1186/s12931-019-1032-z) contains supplementary material, which is available to authorized users.

## Background

Chronic obstructive pulmonary disease (COPD) is characterized by progressive airflow obstruction accompanied by chronic inflammation. It is a major and growing cause of morbidity and mortality worldwide [1]. Although environmental exposures such as cigarette smoking are risk factors, a genetic component to susceptibility has been observed [2–5]. Genomic regions influencing COPD susceptibility have been identified at multiple loci through genome-wide association studies [6–12]. Airway inflammation and remodeling and emphysematous destruction in the lung contribute to disease severity and progression [13, 14], with macrophage activity having an important role [15, 16]. The recapitulation of these gene expression signals in peripheral blood remains elusive. However, gene expression in blood has been used as proxy in identification of COPD subtypes [17], and peripheral blood gene expression underlines the systemic effects of COPD inflammation [18–20].

Several published COPD studies have performed microarray gene expression profiling [21]. Specifically, studies in the airway epithelium have focused on expression changes related to smoking [22, 23] and COPD status [24, 25], including targeted RNA-seq profiling [26]. Studies of gene expression in peripheral blood have also focused on COPD [19, 27, 28] and smoking [29, 30], including RNA-seq profiling [31]. Given the putative role macrophages have in inflammatory lung disease [32], gene expression profiling of these cells has been performed in the context of COPD [33] and smoking [34]. In addition to the airway studies, there have also been several COPD and emphysema gene expression studies involving resected lung tissue [35–39], including RNA-seq profiling in a cohort of males [40] and RNA-seq profiling of early COPD and emphysema in males [41].

Despite the volume of this previous work, the expression signatures for alveolar macrophages, bronchial epithelium, and peripheral blood have not previously been studied within the same population at the same time. However, gene expression in nasal and bronchial brushing samples from the same subjects has been compared [42]. Another study of nasal and bronchial gene expression was performed in independent cohorts [43]. A study of lung tissue, small airway, and peripheral blood gene expression, with tissue samples obtained from separate cohorts, involved both emphysema and lung function phenotypes [44]. Overlapping gene expression signatures have been studied in alveolar macrophages and peripheral monocytes isolated from separate cohorts [45]. Gene expression signatures have been explored across many tissues in the Genotype-Tissue Expression (GTEx) project [46], leveraging network methods to identify tissue-specific gene and transcription factor regulation [47–49] and examining the overall blood-lung gene expression overlap [50].

The foundation of this study is the integration of RNA-seq profiling across three COPD-relevant tissues from the same COPDGene (Genetic Epidemiology of COPD) study subjects, mitigating variation typically seen when studying tissue samples from different subjects. Gene expression in the airway epithelium, alveolar macrophages and whole blood samples were tested for association with measures of lung function, airway disease, emphysema severity and cigarette smoke exposure. Given data across three tissues and 11 phenotype variables, we believed a comprehensive hypothesis could not be the goal. Instead, highlighting private and overlapping gene signatures when present was the more effective approach. Using statistical methods and a gene set enrichment framework, we sought to detect expression signatures across the tissues, highlighting systemic and tissue-specific signatures of lung disease and damage. By integrating these findings with previous COPD lung tissue studies and a recent COPD Genome-wide Association Study (GWAS), we sought to place our results in the context of lung disease biology and shed light on the functional role of genes previously identified at genome-wide significant COPD GWAS loci. Similar integration approaches have been previously applied in COPD [43, 44, 51]. Systems biology has the potential to reveal the molecular architecture of complex traits and disease [52] in part by examining broad biological information rather than individual genomic determinants. We hypothesized that this systems biology study would inform blood biomarker identification, motivate hypotheses regarding the systemic functions of lung disease, and potentially identify novel genes and pathways for COPD and emphysema, as targets for functional, translational and diagnostic studies.

## Methods

### Study subjects and bronchoscopy procedure

Subjects were enrolled in the COPDGene study [53] and participated in the five-year follow-up phase. COPDGene is a longitudinal cohort study that includes non-Hispanic Whites and African Americans enrolled at 21 centers across the United States. The subjects include a small number of non-smokers and more than 10,000 current and former cigarette smokers with a minimum 10 pack-years smoking history. Cases have airflow obstruction (FEV1/FVC < 0.7) and control subjects had normal spirometry (FEV1% predicted ≥80% and FEV1/FVC ≥ 0.7). Subjects returned for the follow-up visit approximately 5 years after enrollment. At this second phase visit, the subjects completed questionnaires and underwent pre- and post-bronchodilator spirometry, volumetric computed tomography (CT) of the chest, and had blood drawn for a complete blood cell count and biomarker studies. Emphysema severity was quantified via image analysis of chest CT data as the percentage of lung voxels below − 950 HU [54].

A single physician performed flexible bronchoscopy on all subjects in the substudy, using intravenous sedation and topical anesthesia. Bronchoalveolar lavage (BAL) was performed in two lung segments with 60 ml of normal saline in each segment, and the BAL fluid obtained from each subject was pooled. Large airway brushings were performed in the right mainstem bronchus and placed in RLT buffer (Qiagen). Whole blood was collected in a PaxGene RNA tube on the day of bronchoscopy. Study subjects provided separate written informed consent for the bronchoscopy study, which was approved by the institutional review board at Partners Healthcare.

### Differential gene expression

We performed differential gene expression (DGE) analysis in each tissue individually using the R/Bioconductor package DESeq2 [55], testing associations between transcript expression levels and lung function, emphysema, smoking and airway disease phenotypes. The base statistical model included the covariates age, sex, race, pack-years of smoking, a categorical variable for smoking status, and RIN (RNA Integrity Number). For analysis of emphysema variables, BMI was included as a covariate. We controlled for cell distribution in peripheral blood using the covariates: white blood cell (WBC) count, and the percentages of neutrophils, lymphocytes, monocytes and eosinophils. A summary of all models is provided in Table 1. Latent effects were addressed using surrogate variables as covariates. These were obtained using the function svaseq in the R/Bioconductor package sva [56]. Prior to svaseq processing, coarse filtering was performed by excluding transcripts with an average count per sample of less than one. Only the surrogate variables lacking a statistically significant association with the phenotype variable of interest were included as covariates. For the differential expression analysis, adjustment for multiple testing controlled for false discovery rate (FDR). In this FDR calculation, the method DESeq2 excludes transcripts with mean normalized counts across all samples that are below a set threshold. By default, this threshold maximizes the number of significant results found at a user-specified FDR. The FDR was chosen to be 10% for this study, as the smaller sample size dictates a value greater than 5%. Results lacking an adjusted *p*-value (NA/not available) are not statistically significant, as they represent genes with mean normalized counts below the threshold. For the continuous variables, the log2 fold change is per unit of change of that variable.

**Table 1:** Models for expression association with outcomes of interest

Phenotype category	Variable of interest	Model
Lung function	COPD case-control	EXP ~ COPD + age + sex + race + pack-years + smoking + RIN + SVs
	FEV1% predicted	EXP ~ FEV1.PP + age + sex + race + pack-years + smoking + RIN + SVs
	FEV1/FVC	EXP ~ FEV1FVC + age + sex + race + pack-years + smoking + RIN + SVs
Smoking	pack-years	EXP ~ pack-years + age + sex + race + RIN + SVs
	Smoking status	EXP ~ smoking + age + sex + race + RIN + SVs
Emphysema	pctEmph	EXP ~ pctEmph + age + sex + race + BMI + pack-years + smoking + RIN + SVs
	perc15	EXP ~ perc15 + age + sex + race + BMI + pack-years + smoking + RIN + SVs
	adj_density	EXP ~ adj_density + age + sex + race + BMI + pack-years + smoking + RIN + SVs
Airway disease	Pi10	EXP ~ Pi10 + age + sex + race + pack-years + smoking + RIN + SVs
	AWT	EXP ~ AWT + age + sex + race + pack-years + smoking + RIN + SVs
	WallAreaPct	EXP ~ WallAreaPct + age + sex + race + pack-years + smoking + RIN + SVs
Models for peripheral blood also included: WBC, and the percentages of neutrophils, lymphocytes, monocytes and eosinophils		
smoking (ordinal variable): 0 non-smoker, 1 former smoker, 2 current smoker		

Abbreviations: SVs = surrogate variables; FEV1 = forced expiratory volume in 1 s; FVC = forced vital capacity; pctEmph = % emphysema; perc15 = 15th percentile of lung density histogram; adj_density = adjusted lung density, sponge model adjustment; Pi10 = SRWA-Pi10 = square root wall area of a hypothetical airway with 10 mm internal perimeter; AWT = airway wall thickness; WallAreaPct = wall area percent; EXP = gene expression values; WBC = white blood cell count

## Results

### Differential gene expression

**Fig. 1:**
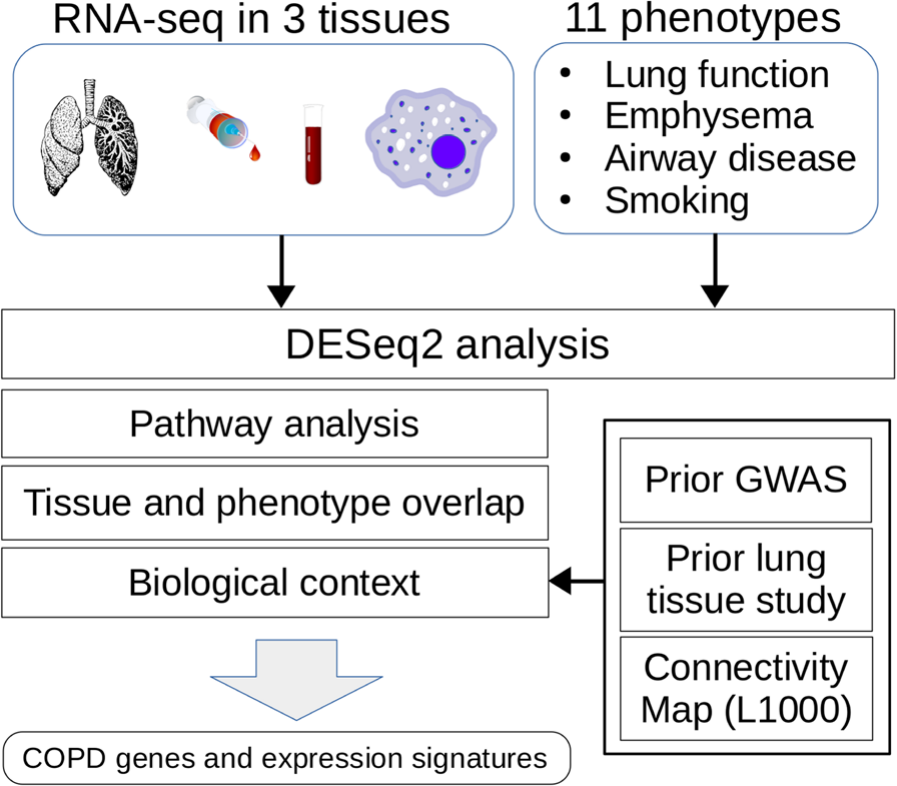
Overview of the study design illustrating the statistical and gene enrichment framework and the tissues (bronchial epithelium, peripheral blood and alveolar macrophages) and the phenotypes investigated. Findings are integrated with prior GWAS, prior lung tissue studies and the Connectivity Map

**Fig. 2:**
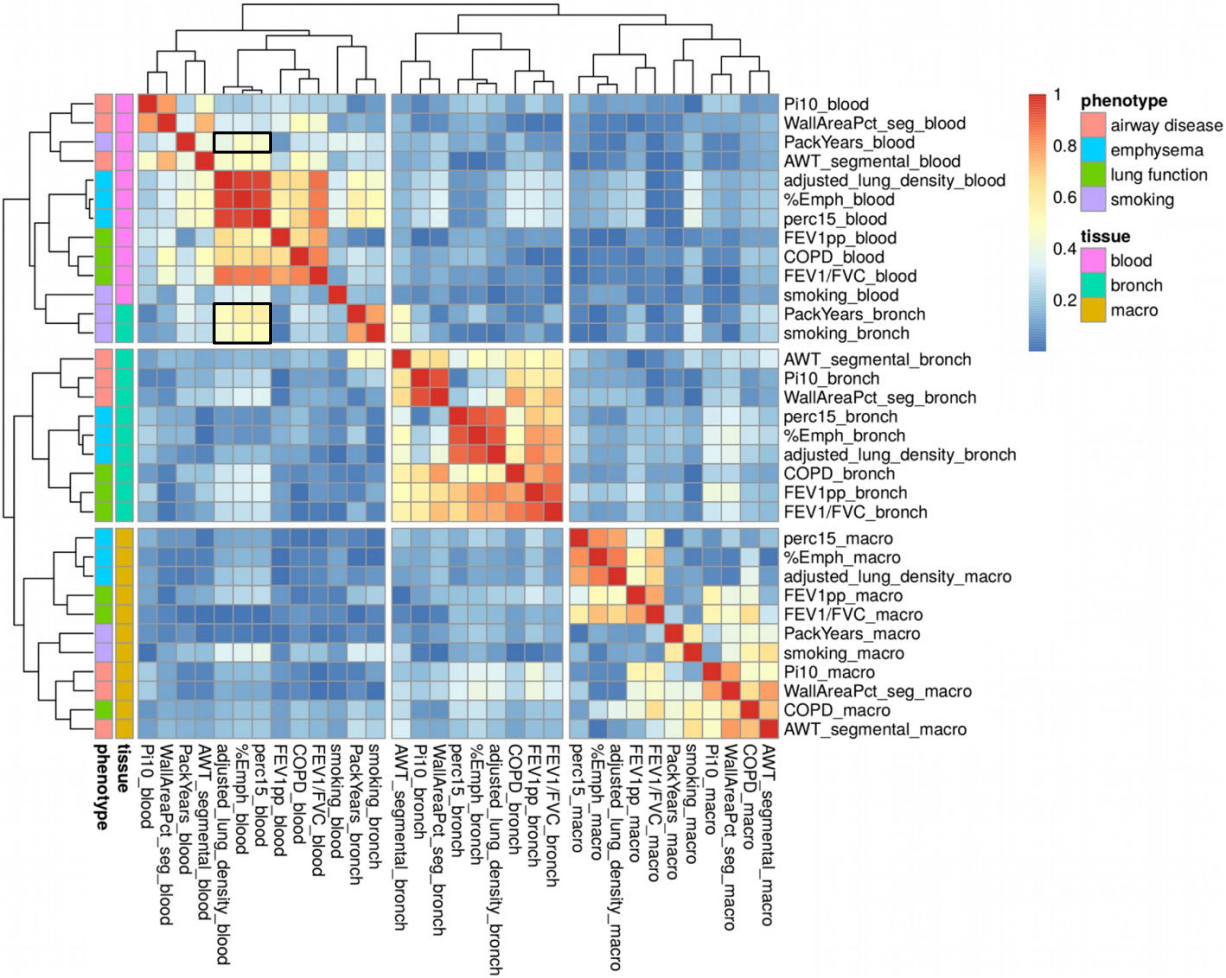
Heatmap of differential gene expression correlation across all analyses. The row and column labels indicate the phenotype variable and the tissue. The results for each analysis were sorted by log2FoldChange and the Spearman correlation was calculated for each pair of results. The absolute value of these correlations is plotted in the heatmap. Clustering by euclidean distance is shown in the dendrograms. The region of correlation between the emphysema signature in blood and the smoking signature in the bronchial epithelium is outlined in the bottom black box (rows 12 & 13; columns 5–7). The region of correlation between the smoking and emphysema signatures in blood is outlined in the top black box (row 3; columns 5–7)

RNA-seq data were available in one or more of the three tissues for 39 subjects, encompassing 94 total samples (Additional file 1: Figure S1). This study focused on the 21 subjects having data available in all three tissues (see Additional file 2: Methods). We performed RNA-seq profiling followed by pathway and enrichment analysis (Fig. 1) on the 63 samples from these 21 subjects (6 COPD cases and 15 controls; Additional file 3: Table S1). We observed greater clustering by tissue than by individual. Emphysema and airway data from CT scans are available for a subset of the subjects. Subjects who never smoked were excluded from analyses, except analyses for the two models assessing the impact of smoking. Association between gene expression and 11 phenotype variables (Table 1) was tested in each tissue. We viewed the overlapping gene expression signatures of all results included in the DESeq2 FDR calculations (Additional file 3: Table S2), using a correlation heatmap (Fig. 2). Within this heatmap, we observed clustering by both tissue and phenotype, presenting as blocks of higher correlation. Specifically, the clustering by phenotype variable (mirroring our phenotype category grouping) is nested within the clustering by tissue. However, some smoking results in bronchial epithelium grouped with the peripheral blood module (top left in Fig. 2). Delving into this heatmap reveals this clustering is driven by the correlation between the emphysema signature in blood and the smoking signature in the bronchial epithelium (rows 12 & 13; columns 5–7 within black outline, Fig. 2). These signatures reside within the complete differential expression results for the specific tissue and phenotype. Another feature of note is the clustering of the smoking signature in blood with the airway-disease signature in blood, driven by the correlation between the smoking and emphysema signatures in blood (row 3; columns 5–7 within black outline, Fig. 2).

**Fig. 3:**
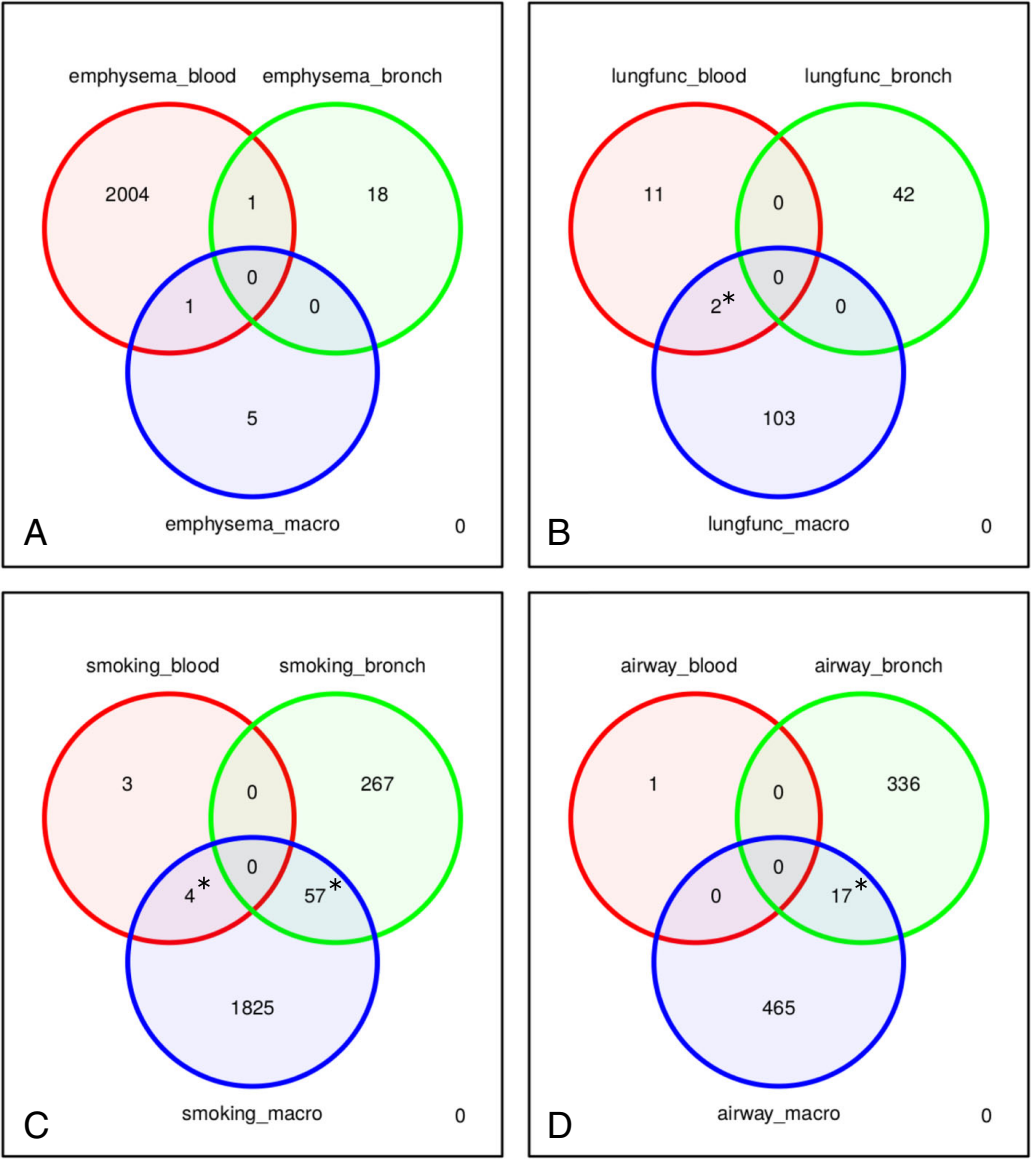
Venn diagrams of the combined DESeq2 results intersected across tissue for the four phenotype categories (**a**. emphysema, **b**. lung function, **c**. smoking status, **d**. airway disease); an asterisk denotes significant overlap (*p* < 0.01)

The number of significant (q-value < 0.1) results across all 33 analyses (11 variables across 3 tissues) varied from zero to 1886 (Additional file 3: Table S3). Since log2 fold change depends on the units for each variable, we did not apply a set fold change filter when determining significance. Of these 33 sets of results, 26 contained at least one significant result (Additional file 3: Tables S4-S6); the intersection pattern across the results was complex and mixed (Additional file 1: Figure S2). Next, to enhance the gene expression signal for each phenotype, we combined the significant (q-value < 0.1) genes across the phenotype variables in each of the four phenotype categories (lung function, airway disease, emphysema severity and cigarette smoke exposure), retaining the unique genes in each category. Venn diagrams highlight any cross-tissue intersections of these combined results (Fig. 3, Additional file 3: Table S7). We performed hypergeometric tests of gene enrichment across the tissues within each phenotype category (Fig. 3 and Additional file 3: Table S8). The backgrounds in these tests were the unique common genes across each pair of tissues having an average of at least two reads in each tissue. We observed statistically significant enrichment primarily across bronchial epithelium and alveolar macrophages. Although there were statistically meaningful overlaps in the macrophage and blood sets, the number of intersecting genes was less than five. We did not observe genes differentially expressed in all three tissues for any of the four phenotype categories.

### Enrichment and signature analyses

**Fig. 4:**
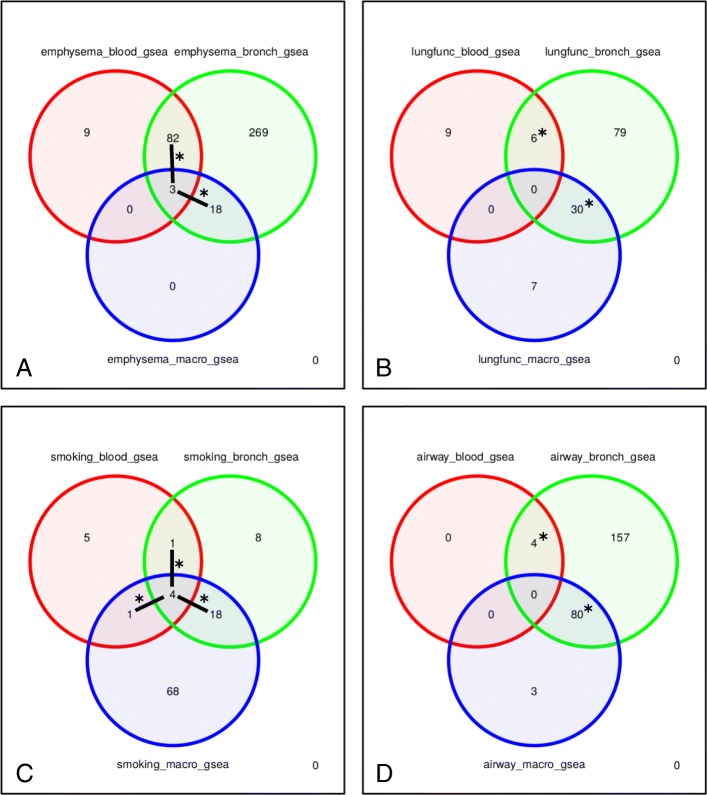
Venn diagrams of the overlap across tissue of the combined gene set enrichment results for the four phenotype categories (**a**. emphysema, **b**. lung function, **c**. smoking status, **d**. airway disease); an asterisk denotes significant overlap (p < 0.01) and lines join non-zero counts contributing to a significant overlap

We performed pathway analyses on the results for each phenotype association using gene set enrichment, and combined the significant (q-value < 0.05) findings across the four phenotype categories (Fig. 4, Additional file 3: Table S9). In contrast to the results for individual genes, we did observe pathway overlaps across all three tissues for emphysema and smoking. We performed hypergeometric tests of pathway enrichment across the tissues within each phenotype category (Fig. 4 and Additional file 3: Table S8). The background for these tests were the set of pathways tested. We observed statistically significant enrichment across two or more pairs of tissues for each of the phenotype categories. To provide a graphical pathway summary, we created an Enrichment Map in Cytoscape using the overlapping pathways for blood and bronchial epithelium in emphysema (Additional file 1: Figure S3). We observed network modules characterized by metabolic, cancer, and immune signaling pathways, with smaller groups containing adherens junction and focal adhesion pathways.

**Fig. 5:**
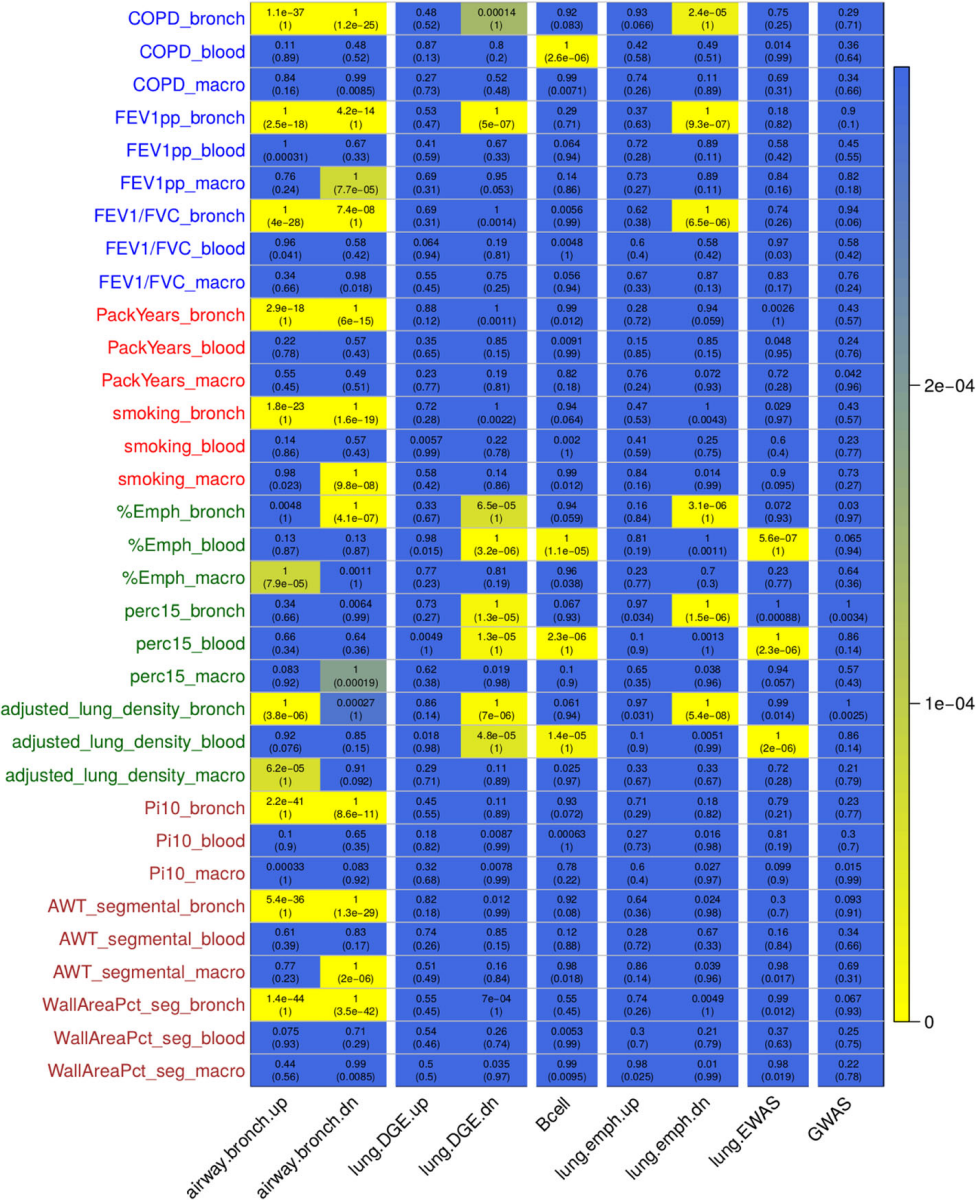
Heatmap summary of *p*-values from gene set enrichment tests using a set of significant (q-value < 0.1) airway disease results in the bronchial epithelium, and findings from previous GWAS and lung tissue studies. The top p-value corresponds to enrichment test in the up-regulated genes and the bottom (p-value) refers to enrichment in down-regulated genes. The row labels are color-coded by phenotype category (blue = lung function, red = smoking; green = emphysema, brown = airway)

To further assess disease relevance, we performed gene set enrichment tests to compare the differential gene expression signatures with findings from three previous studies (see Additional file 2: Methods). The set of significant (q-value < 0.1) genes from the analysis of airway phenotypes in the bronchial epithelium was included as a disease tissue reference. We summarized these enrichment findings for both up- and down-regulated genes in a *p*-value heatmap (Fig. 5). From the heatmap, we observed enrichment of the bronchial epithelium airway-disease genes in macrophage results across all four phenotype categories. Within the integration with previous studies (lung tissue gene expression, lung tissue DNA methylation and COPD GWAS), we observed enrichment of lung tissue COPD and emphysema genes in our bronchial epithelium results for both lung function and emphysema. The down-regulated lung tissue genes were found enriched in the genes up-regulated in bronchial epithelium by disease status. We also observed enrichment of our previously published COPD-associated B cell lung tissue expression module [38] and lung tissue DNA methylation genes [57] within the emphysema results for peripheral blood. We did not find enrichment for the lung emphysema genes in these phenotype variables nor did we find enrichment of the B cell module in the bronchial epithelium or macrophage results. We extracted the significant (q-value < 0.1) differential expression genes intersecting the external gene sets (Additional file 3: Table S10). These enrichment experiments provided lung disease context by linking to gene expression and epigenetic signatures of COPD in lung tissue.

We queried the Connectivity Map (CMap CLUE) using our bronchial epithelium results for airway disease. The perturbagens of interest have negative scores, as these signatures demonstrate reversal of disease severity for both the A549 cell line (Additional file 3: Table S11) and HCC515 cell line (Additional file 3: Table S12). The top chemical pertubagen from the A549 results was lomerizine and the top result for HCC515 was ephedrine.

## Discussion

We integrated RNA-sequencing across three matched COPD-relevant tissues using tests of association with lung function, airway disease, emphysema severity and cigarette smoke exposure, and a gene set enrichment framework. This has revealed expression signatures across the tissues in the context of each phenotype, highlighting systemic and tissue-specific signatures and pathways of lung disease and damage. We did not observe any genes differentially expressed across all three tissues. However, we did find pathways overlapping the three tissues in emphysema and smoking. Disease relevance and biology were elucidated through integration with previous COPD lung tissue studies and a recent COPD GWAS.

### Replication of airway differential gene expression

Our top two results from the differential expression analysis of smoking status in the bronchial epithelium were *CYP1A1* (cytochrome P450 family 1 subfamily A member 1) and *CYP1B1* (cytochrome P450 family 1 subfamily A member 1). These replicate previous findings in studies of smoking in the airway [23, 58] and oral mucosa [59], with *CYP1B1* also identified in the lung [60]. Significant in our analysis of smoking was *AHRR* (aryl-hydrocarbon receptor repressor), previously found to be differentially expressed by smoking status in lung tissue [60] and in the oral mucosa [59]. Both *CYP1B1* and *AHRR* were also significant in our analysis of smoking status in macrophages, and Poliska et al. also found *CYP1B1* correlated with COPD status in alveolar macrophages [45].

In our bronchial epithelium analysis of airway disease, the genes *CLDN10* (claudin 10), *TMEM2* (*CEMIP2* - cell migration inducing hyaluronidase 2) and *ALDH1A3* (aldehyde dehydrogenase 1 family member A3) were significant across the three airway-disease variables. *CLDN10* is believed to have a role in idiopathic pulmonary fibrosis (IPF) progression [61]. A gene-by-environmental tobacco smoke interaction study on the level of FEV1 identified a locus intronic to the gene *TMEM2* [62] and *TMEM2* was previously associated with lung function in the small airway [44]. Last, the gene *ALDH1A3* was found to be differentially expressed by smoking status in both the bronchial and nasal epithelium [42].

The top gene in our bronchial epithelium analysis of percent emphysema was *APOD* (apolipoprotein D), a gene found differentially expressed in a study of emphysema severity and bronchiolitis [37]. The second gene in this emphysema analysis was *CYP2A6* (cytochrome P450 family 2 subfamily A member 6) from a locus previously identified in GWAS of smoking behavior [63] and COPD [7]. These replications suggest a link to smoking-related lung disease and progression, with relevance throughout the respiratory tract.

### Pathways overlap across tissues

We observed a mixed and complex overlap pattern of significant genes across all differential expression results. To better glean information from the overlaps, we focused on private and cross-tissue signatures. We combined the differential expression and pathway results across phenotype variables, based on our observations of clustering by phenotype categories in the correlation heatmap. In this context, we observed statistically significant enrichment primarily across the bronchial epithelium and alveolar macrophages. We did not observe genes differentially expressed in all three tissues for any of the four phenotype categories. However, for emphysema and smoking we did observe pathway overlaps across all three tissues. We also observed statistically significant pathway overlaps across pairs of tissues in each of the four phenotype categories. In emphysema, the pathways at the three-tissue intersection were related to hemostasis and immune signaling, both markers of systemic inflammation. The three-tissue overlap for smoking included amyloid and telomere related pathways. This is concordant with observations of amyloids as putative biomarkers of systemic inflammation and COPD [64] and the association between lung disease, lung aging, and telomere length [65].

In addition to the three-tissue intersections, the robust two-tissue pathway overlap in airway disease for the bronchial epithelium and macrophages appears to be localized with signatures of oxidative stress, highlighted by enrichment of nonsense mediated decay and metabolic pathways. The cell-cycle pathways also present in this overlap are suggestive of cellular senescence mechanisms [66, 67], particularly given the findings in emphysema for these cells [68]. A differentially expressed gene observed at this intersection and the bronchial epithelium and macrophage intersection for smoking was *SCGB1A1* (secretoglobin family 1A member 1). This gene is expressed at high levels in the lung and encodes for CC16 (Club Cell Secretory Protein) a blood biomarker of COPD [69, 70].

Another significant pathway overlap was observed between blood and bronchial epithelium in emphysema, characterized by clusters of metabolic, cancer, and immune signaling pathways, with adherens junction and focal adhesion pathways also present. These pathways highlight signals of structural damage and systemic immune response in airway disease and emphysema [14, 71]. The significantly differentially expressed gene at this intersection was *FCN1* (ficolin 1), a gene found to be differentially expressed in peripheral blood in mild IPF [72]. In addition, functional polymorphic sites in the promoter region of *FCN1* regulate ficolin-1 expression and influence outcomes during systemic inflammation [73].

### Airway signatures overlap in blood and recapitulate in lung

We observed clustering of the smoking signature in the bronchial epithelium with the smoking signature in blood in our differential expression correlation heatmap, brought about by the relationship between the emphysema signature in blood and the smoking signature in the bronchial epithelium. We also observed clustering of the smoking signature in blood with the airway-disease signature in blood, owing to the correlation between the smoking and emphysema signatures in blood. Taken together, these suggest a common and systemic marker of emphysema with a gene expression signature of smoke-induced damage [18, 44, 74].

We integrated our differential expression results with findings from previous COPD studies, along with the significant bronchial epithelium results in airway disease. We observed enrichment of the bronchial epithelium airway-disease genes in macrophage results across all four phenotype categories. This was in line with the findings when we intersected the significant genes and pathways for these tissues. Lung tissue COPD and emphysema genes were enriched in our bronchial epithelium results for both the lung function and emphysema phenotype categories, demonstrating disease relevance in lung tissue. We found that the down-regulated lung tissue genes were found enriched in the genes up-regulated in the bronchial epithelium by disease status. Although not an equivalent comparison, this finding is similar in nature to that of Obeidat et al., [44], where the lung tissue and blood gene expression directionality was opposite across their two tissue cohorts for a majority of the genes of interest.

Within the emphysema results for peripheral blood, we also observed enrichment of the COPD-relevant lung tissue B cell expression module [38] and DNA methylation [57] gene sets. The direction of effect for these enrichments were concordant with respect to disease status. The methylation directionality relationship is more difficult to resolve given the various gene regulation mechanisms [75]. We did not find enrichment for the lung emphysema genes in these phenotype variables. Overall, this suggests a systemic B cell signature observed previously in the lung [38], recapitulated here in peripheral blood. The significant gene at B cell module intersection with the bronchial epithelium results for COPD was *CD28* (CD28 molecule). This gene may play a role in immunologic senescence [71] and COPD inflammation [76], owing to its role as a co-stimulatory molecule, constitutively expressed by naïve T cells and required for full activation (and survival) of T cells.

### Reversal of bronchial epithelium disease signature

Using Connectivity Map, we identified perturbing compounds that produce gene expression signatures in two lung cell lines opposing the disease gene expression signature we observed in the bronchial epithelium. The top chemical pertubagen from the A549 results was lomerizine, a calcium channel blocker, suggesting potential drug repurposing. Others on the list include glucocorticoid receptor agonists, used in the treatment of inflammatory lung diseases [77] through their activation of specific glucocorticoid receptor mechanisms. Among the HCC515 compounds, fluticasone is a current therapeutic for treatment of respiratory disease [78], and as the top result for HCC515, ephedrine is a known bronchodilator.

Some limitations to the current study involve blood and bronchial epithelium cellular heterogeneity. We have partially addressed the heterogeneity in blood using the measured leukocyte counts. However, remaining variation (e.g. lymphocyte composition) may influence the gene expression signatures, as *GPR15* was differentially expressed in our smoking analysis in blood and was found to be expressed in a T cell dependent manner with cigarette smoking [79]. Single cell or single cell type sequencing will better resolve specific gene expression signatures. We have not addressed the polarization of the alveolar macrophages, that increases with COPD severity and cigarette smoke exposure [80]. The study of early and intermediate phenotypes of COPD would help to link the temporal changes in the tissue gene expression overlap with disease progression, as would longitudinally repeated gene expression experiments. Last, despite the use of RNA-seq to improve the resolution of gene expression signatures and use of gene set enrichment to extract signals from all results, our sample size does limit our power to detect these signatures. Given this limited power, our focus was not on the identification of specific biomarkers. Future work will involve larger study cohorts with greater power to also resolve individual biomarkers.

## Conclusions

In this integrative genomics study, we have performed RNA-seq profiling of gene expression in three matched COPD-relevant tissues. Using statistical and gene set enrichment methods, we have identified overlapping differentially expressed genes and pathways across the tissues, providing lung disease biomarker insight. We observed no common genes across all three tissues. However, we did observe shared pathways across all three. By integrating the gene expression profiles with previous COPD findings to provide additional disease context, we identified a lung disease signature in our emphysema results in the bronchial epithelium and peripheral blood, while also suggesting recapitulation of a systemic B cell lung signature in peripheral blood. Together this hints that peripheral blood has the potential to capture relevant lung pathobiology. Connectivity Map provided some translational context, identifying known and putative compounds that elicit a gene expression signature in lung cell lines that opposes the disease signature we observed in the bronchial epithelium.

## Additional files

Additional file 1: Supplemental Figures: Additional figures to support the findings of this study. (PDF 780 kb)

Additional file 2: Supplemental Methods. Additional detail regarding the methods in this study. (PDF 56 kb)

Additional file 3: Supplemental Tables: Additional tables to support the findings of this study. **Table S4.** Differential gene expression results in bronchial epithelium. **Table S5.** Differential gene expression results in alveolar macrophages. **Table S6.** Differential gene expression results in peripheral blood. (ZIP 674 kb)

## Abbreviations

*AHRR*: Aryl-hydrocarbon receptor repressor
*ALDH1A3*: Aldehyde dehydrogenase 1 family member A3
*APOD*: Apolipoprotein D
BAL: Bronchoalveolar Lavage
CC16: Club Cell Secretory Protein
*CLDN10*: Claudin 10
CMap: Connectivity Map
COPD: Chronic Obstructive Pulmonary Disease
COPDGene: Genetic Epidemiology of COPD
CT: Computed Tomography
*CYP1A1*: Cytochrome P450 family 1 subfamily A member 1
*CYP1B1*: Cytochrome P450 family 1 subfamily A member 1
*CYP2A6*: Cytochrome P450 family 2 subfamily A member 6
DGE: Differential Gene Expression
*FCN1*: Ficolin 1
FDR: False Discovery Rate
GTEx: Genotype-Tissue Expression
GWAS: Genome-wide Association Study
IPF: Idiopathic Pulmonary Fibrosis
RIN: RNA Integrity Number
*SCGB1A1*: Secretoglobin family 1A member 1
*TMEM2*: *CEMIP2* - cell migration inducing hyaluronidase 2
WBC: White Blood Cell
