# Supplementary material for: RNA-sequencing across three matched tissues reveals shared and tissue-specific gene expression and pathway signatures of COPD
Source: Respir Res. 2019 Apr 2;20:65. doi: 10.1186/s12931-019-1032-z (PMC6446359; doi:10.1186/s12931-019-1032-z)
Supplement: Supplementary file 1 — Supplemental Figures: Additional figures to support the findings of this study. (PDF 780 kb) [file 12931_2019_1032_MOESM1_ESM.pdf]

RNA-sequencing across three matched tissues reveals shared and tissue-specific gene expression and pathway signatures of COPD

Jarrett D. Morrow[1], Robert P. Chase[1], Margaret M. Parker[1], Kimberly Glass[1], Minseok Seo[1], Miguel Divo[2], Caroline A. Owen[2], Peter Castaldi[1], Dawn L. DeMeo[1,2], Edwin K. Silverman[1,2], Craig P. Hersh[1,2]

1. Channing Division of Network Medicine, Brigham and Women's Hospital, Boston, MA, 02115

2. Division of Pulmonary and Critical Care Medicine, Brigham and Women's Hospital, Boston, MA, 02115

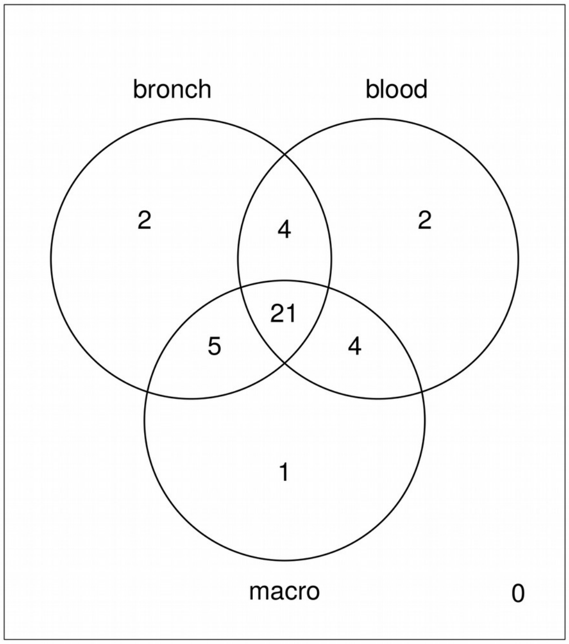

Figure S1. Summary of the RNA-seq data available across subjects and tissues (39 subjects, 94 total samples).

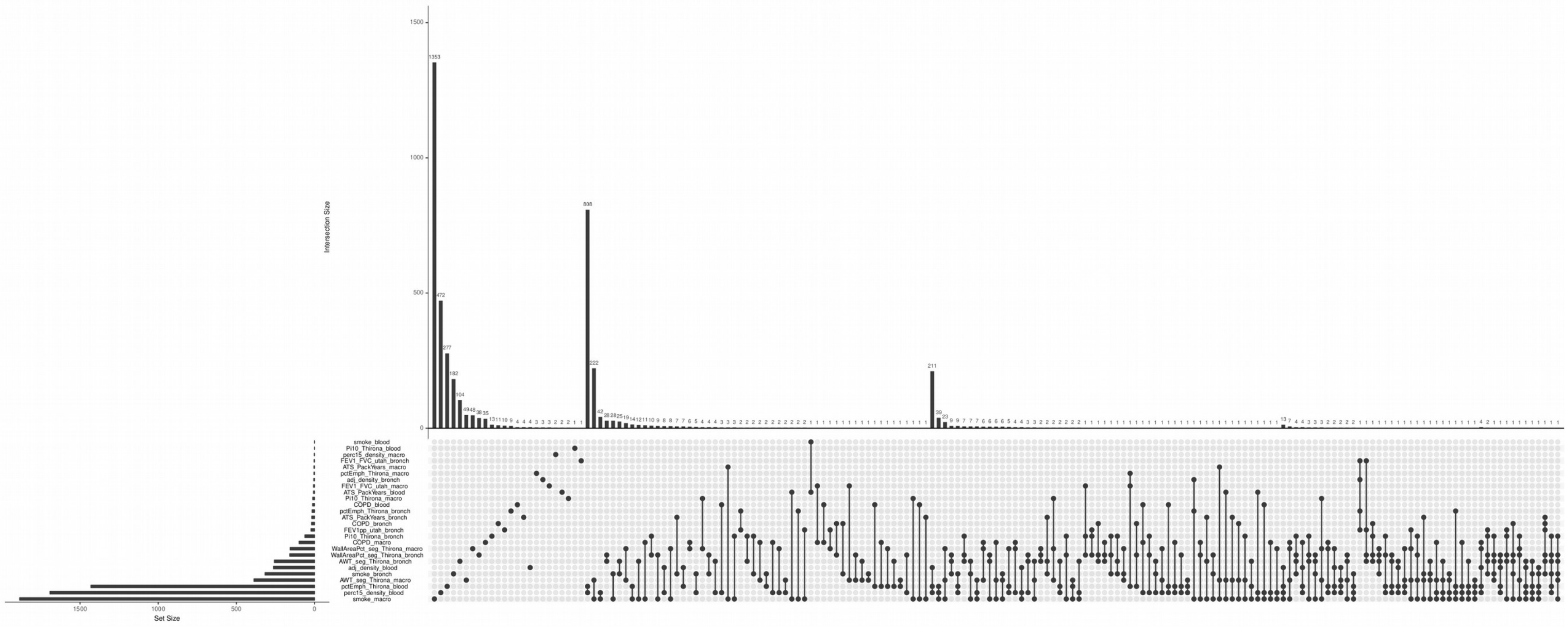
Intersection Size
Set Size
smoke_blood
Pi10_Thirona_blood
perc15_density_macro
FEV1_FVC_utah_bronch
ATS_PackYears_macro
pctEmph_Thirona_macro
adj_density_bronch
FEV1_FVC_utah_macro
ATS_PackYears_blood
Pi10_Thirona_macro
COPD_blood
pctEmph_Thirona_bronch
ATS_PackYears_bronch
COPD_bronch
FEV1pp_utah_bronch
Pi10_Thirona_bronch
COPD_macro
WallAreaPct_seg_Thirona_macro
WallAreaPct_seg_Thirona_bronch
AWT_seg_Thirona_bronch
adj_density_blood
smoke_bronch
AWT_seg_Thirona_macro
pctEmph_Thirona_blood
perc15_density_blood
smoke_macro

Figure S2. UpSet plot of the intersection of DESeq2 results for each of the three tissues and across the 11 phenotype variables. Each column provides a Venn diagram type intersection. Horizontal bars (Set Size) represent the number of differentially expressed genes. Single solid dots represent genes that do not intersect with any other set, and the solid dots connected by lines represent intersections. The vertical bars (Intersection Size) are proportional to the total number of genes for the solid dot(s) below them. For any given set (any row), the total number of differentially expressed genes may be determined by summing across all columns containing a solid dot.

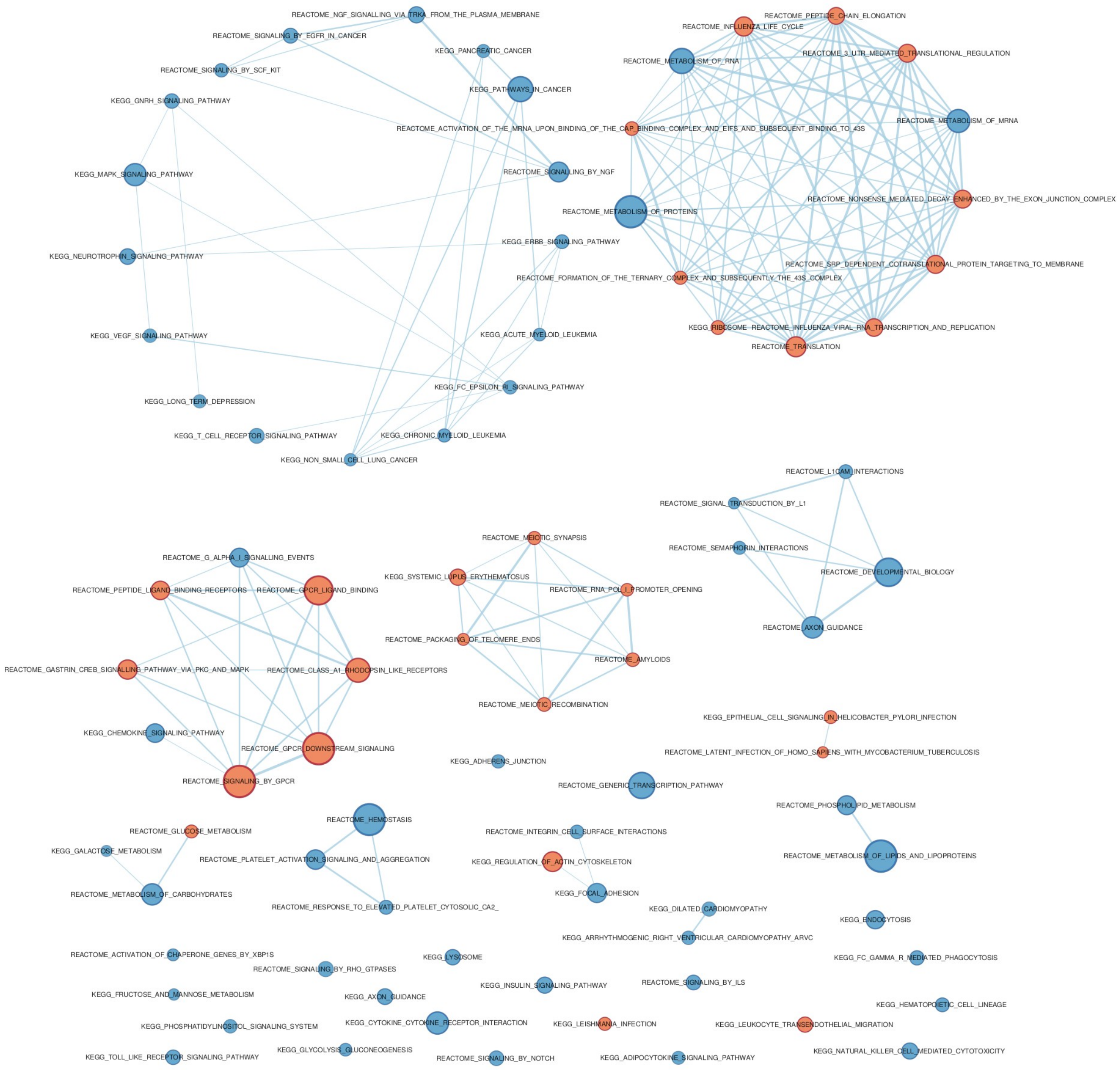

Figure S3. Overlapping pathways from blood and the bronchial epithelium in emphysema
