# Supplementary material for: RNA-sequencing across three matched tissues reveals shared and tissue-specific gene expression and pathway signatures of COPD
Source: Respir Res. 2019 Apr 2;20:65. doi: 10.1186/s12931-019-1032-z (PMC6446359; doi:10.1186/s12931-019-1032-z)
Supplement: Supplementary file 2 — Supplemental Methods. Additional detail regarding the methods in this study. (PDF 56 kb) [file 12931_2019_1032_MOESM2_ESM.pdf]

RNA-sequencing across three matched tissues reveals shared and tissue-specific gene expression and pathway signatures of COPD

Jarrett D. Morrow[1], Robert P. Chase[1], Margaret M. Parker[1], Kimberly Glass[1], Minseok Seo[1], Miguel Divo[2], Caroline A. Owen[2], Peter Castaldi[1], Dawn L. DeMeo[1,2], Edwin K. Silverman[1,2], Craig P. Hersh[1,2]

1. Channing Division of Network Medicine, Brigham and Women's Hospital, Boston, MA, 02115

2. Division of Pulmonary and Critical Care Medicine, Brigham and Women's Hospital, Boston, MA, 02115

Supplemental Methods

Macrophage isolation

BAL (Bronchoalveolar lavage) fluid was filtered with a sterile 70 μm cell strainer to remove mucus. After centrifugation, the BAL cells were resuspended in DMEM (Dulbecco's Modified Eagle's medium) containing 10% fetal bovine serum and antibiotics, and counted with a Neubauer chamber. A cytocentrifuge preparation of the cells was then stained with modified Wright's stain, and differential cell counts were performed to assess alveolar macrophage purity. To obtain a purified set of macrophages by using cellular adherence, the BAL leukocytes were incubated in 24-well tissue culture plates for two hours at 37ºC in a humidified atmosphere of 5% CO2. Contaminating PMNs and lymphocytes (which are non-adherent cells) were removed by washing the wells with medium, and the adherent macrophages were lysed for gene expression.

RNA sequencing

For each tissue, total RNA was extracted using the AllPrep kit (Qiagen, Valenica, CA). RNA quality was assessed on a BioAnalyzer (Agilent, Santa Clara, CA). Extracted RNA samples with RNA integrity number (RIN) greater than six and a concentration greater than or equal to 20 μg/ul were sequenced. Libraries were QCed by quantification with picogreen, size analysis on an Agilent Bioanalyzer (Agilent, Santa Clara, CA) and qPCR quantitation against a standard curve. Paired end reads with nominal 75 bp length were generated on an Illumina HiSeq 2500 flow cell. Sequencing was performed to an average depth of 20 million reads.

The quality control pipeline included FastQC (1) and RNA-SeQC (2). Adapter trimming was performed using Skewer (3). STAR aligner version 2.4.0h (4) was used to map the reads to the GRCH38 genome reference. The Python framework HTSeq was used to produce gene-level counts (5) with the Ensembl version 81 gene annotation (6). The samples retained for analysis each had more than 7 million total reads, and greater than 70% of reads mapped to the reference genome, with less than 10% of R1 reads in the sense orientation. One subject was excluded due to lack of concordance between genotype calls in RNA sequencing and prior DNA genotyping data. Using the Y-chromosome expression data, concordance of sex was confirmed for each subject. Following this quality-assessment, samples from all three tissues were available for 21 subjects. Only data for genes mapped to autosomal chromosomes were retained, leaving counts for 54,243 of the 65,988 transcripts available for analysis. These gene expression data have been deposited in Gene Expression Omnibus (GEO accession GSE124180).

Differential gene expression plots

We viewed the summary and intersection of these statistically significant findings using an UpSet plot (7). UpSet plots are akin to Venn diagrams that can effectively present intersections across a large number of gene sets (8),

here illustrating complex overlap patterns across the differential gene expression (DGE) results. To extract and highlight more subtle and overall shared gene expression signatures across tissues and variables, we observed the correlation between the results for each tissue and phenotype variable. We sorted the results for each DESeq2 set by log2FoldChange and excluded genes not found in any of the results; NAs present when results for a gene not available in a particular set of results. The Spearman correlation was calculated for each pair of results (each result labeled: phenotype_tissue). We summarized the absolute value of these correlations using the R package pheatmap. This DGE correlation plot includes a clustering by euclidean distance, presented as dendrograms on the axes.

Gene set enrichment

We performed pathway analyses of the DGE results using gene set enrichment via the R package gage (9). The gage (Generally Applicable Gene-set Enrichment) strategy for gene expression analysis is to reveal pathway knowledge using sets of related genes, and is not limited by datasets with different sample sizes, experimental designs and profiling techniques. For these analyses, the DESeq2 results were sorted by the log2FoldChange value, providing a significance ranking with a sign for DGE direction. The KEGG and Reactome gene set databases were used to provide biological insight. Using these databases, pathway enrichment results with FDR q-value less than 0.05 using the Benjamini and Hochberg method were considered significant. Enrichment of the sets within both up- (greater) and down-regulated (less) genes was considered. We formatted pathway enrichment results for use with the Cytoscape plugin Enrichment Map (10). Enrichment Map presents the relationships between the individual pathway results in a network context (11).

Gene sets from previous studies were used to provide disease context in the gene set enrichment analyses. The first two of these sets were comprised of differentially expressed genes by COPD status and by emphysema severity (Low attenuation areas at -950 HU and 15th percentile of the lung density histogram) in our previous lung tissue study (204 and 255 genes, respectively) (8). These sets were further parsed into up- and down-regulated genes by disease status and severity (COPD: 63 up & 141 down; emphysema: 103 up & 152 down). The third set is a co-expression module from the same study (8). This module was associated with COPD in a lung-tissue co-expression analysis and was enriched for B cell pathways, sharing seventeen genes with a mouse smoking model (12) and twenty genes with previous emphysema studies (13, 14). The fourth consists of genes with CpG sites differentially methylated by COPD status in lung tissue (344 genes) (15). To capture greater biological significance, the mean difference in methylation was required to be greater than 5%, in addition to meeting an FDR < 5%. The fifth gene set was a list of 155 genes within loci identified in a recent large GWAS of COPD and lung function (16). The set of significant (FDR q-value < 0.1) genes from the analysis of airway phenotypes in the bronchial epithelium was included. This set was created by combining results from the three airway disease variables and was parsed by disease severity into up- and down-regulated genes, creating two additional gene sets. We used the Bonferroni

method to correct for multiple testing. Significance was obtained with p < 0.05/(33 analyses x (7 ext. gene sets + 2 ref. gene sets = 9) enrichment tests) = 0.00017.

Connectivity Map

We used the ConnectivityMap within the CLUE platform to identify gene signatures shared between our bronchial epithelium results for the airway disease phenotypes and the database of human cell transcriptional responses to chemical perturbation (L1000 profiles) (17). Sets of significant up-regulated genes (positive log2FoldChange) were created for each of the three airway disease variables. The threshold for inclusion was FDR q-value < 0.05 instead of q-value < 0.1, as this produced a gene count below the 150-gene limit for a CMap query. The set of unique genes in the combination of the three sets was submitted to CMap, combined with a set of down-regulated (negative log2FoldChange) genes produced in the same manner. The perturbagens of interest have negative scores, as these signatures demonstrate reversal of disease severity. Data from the A549 (human non-small cell lung carcinoma) and HCC515 (human non-small cell lung adenocarcinoma) cell lines were the focus. Obtaining a score of 95 indicates that only 5% of reference gene sets demonstrated stronger connectivity to the perturbagen than the query, perhaps suggesting consideration for hypothesis generation. Results for both individual perturbagens and the CMap activity classes were observed, informing specific and general hypothesis options.

References

1. Andrews S. *Fastqc: A Quality Control Tool For High Throughput Sequence Data.* 2010. at <https://www.bioinformatics.babraham.ac.uk/projects/fastqc/>.
2. DeLuca DS, Levin JZ, Sivachenko A, Fennell T, Nazaire M-D, Williams C, Reich M, Winckler W, Getz G. RNA-SeQC: RNA-seq metrics for quality control and process optimization. *Bioinformatics* 2012;28:1530–1532.
3. Jiang H, Lei R, Ding S-W, Zhu S. Skewer: a fast and accurate adapter trimmer for next-generation sequencing paired-end reads. *BMC Bioinformatics* 2014;15:182.
4. Dobin A, Davis CA, Schlesinger F, Drenkow J, Zaleski C, Jha S, Batut P, Chaisson M, Gingeras TR. STAR: ultrafast universal RNA-seq aligner. *Bioinformatics* 2013;29:15–21.
5. Anders S, Pyl PT, Huber W. HTSeq--a Python framework to work with high-throughput sequencing data. *Bioinformatics* 2015;31:166–169.
6. Kersey PJ, Allen JE, Armean I, Boddu S, Bolt BJ, Carvalho-Silva D, Christensen M, Davis P, Falin LJ, Grabmueller C, Humphrey J, Kerhornou A, Khobova J, Aranganathan NK, Langridge N, Lowy E, McDowall MD, Maheswari U, Nuhn M, Ong CK, Overduin B, Paulini M, Pedro H, Perry E, Spudich G, Tapanari E, Walts B, Williams G, Tello–Ruiz M, *et al.* Ensembl Genomes 2016: more genomes, more complexity. *Nucleic Acids Research* 2016;44:D574–D580.
7. Lex A, Gehlenborg N, Strobelt H, Vuillemot R, Pfister H. UpSet: Visualization of Intersecting Sets. *IEEE Transactions on Visualization and Computer Graphics* 2014;20:1983–1992.
8. Morrow JD, Zhou X, Lao T, Jiang Z, DeMeo DL, Cho MH, Qiu W, Cloonan S, Pinto-Plata V, Celli B, Marchetti N, Criner GJ, Bueno R, Washko GR, Glass K, Quackenbush J, Choi AMK, Silverman EK, Hersh CP. Functional interactors of three genome-wide association study genes are differentially expressed in severe chronic obstructive pulmonary disease lung tissue. *Scientific Reports* 2017;7:44232.
9. Luo W, Friedman MS, Shedden K, Hankenson KD, Woolf PJ. GAGE: generally applicable gene set enrichment for pathway analysis. *BMC Bioinformatics* 2009;10:161.
10. Merico D, Isserlin R, Stueker O, Emili A, Bader GD. Enrichment map: a network-based method for gene-set enrichment visualization and interpretation. *PloS one* 2010;5:e13984–e13984.

11. Morrow JD, Qiu W, Chhabra D, Rennard SI, Belloni P, Belousov A, Pillai SG, Hersh CP. Identifying a gene expression signature of frequent COPD exacerbations in peripheral blood using network methods. *BMC Medical Genomics* 2015;8:1.
12. Lao T, Glass K, Qiu W, Polverino F, Gupta K, Morrow J, Mancini JD, Vuong L, Perrella MA, Hersh CP, Owen CA, Quackenbush J, Yuan G-C, Silverman EK, Zhou X. Haploinsufficiency of Hedgehog interacting protein causes increased emphysema induced by cigarette smoke through network rewiring. *Genome Medicine* 2015;7:12.
13. Faner R, Cruz T, Casserras T, López-Giraldo A, Noell G, Coca I, Tal-Singer R, Miller B, Rodriguez-Roisin R, Spira A, Kalko SG, Agusti A. Network Analysis of Lung Transcriptomics Reveals a Distinct B Cell Signature in Emphysema. *Am J Respir Crit Care Med* 2016;193:1242–1253.
14. Campbell JD, McDonough JE, Zeskind JE, Hackett TL, Pechkovsky DV, Brandsma C-A, Suzuki M, Gosselink JV, Liu G, Alekseyev YO, Xiao J, Zhang X, Hayashi S, Cooper JD, Timens W, Postma DS, Knight DA, Lenburg ME, Hogg JC, Spira A. A gene expression signature of emphysema-related lung destruction and its reversal by the tripeptide GHK. *Genome Medicine* 2012;4:67.
15. Morrow JD, Cho MH, Hersh CP, Pinto-Plata V, Celli B, Marchetti N, Criner G, Bueno R, Washko G, Glass K, Choi AMK, Quackenbush J, Silverman EK, DeMeo DL. DNA methylation profiling in human lung tissue identifies genes associated with COPD. *Epigenetics* 2016;11:730–739.
16. Sakornsakolpat P, Prokopenko D, Lamontagne M, Reeve NF, Guyatt AL, Jackson VE, Shrine N, Qiao D, Bartz TM, Kim DK, Lee MK, Latourelle JC, Li X, Morrow JD, Obeidat M, Wyss AB, Zhou X, Bakke P, Barr RG, Beaty TH, Belinsky SA, Brusselle GG, Crapo JD, Jong K de, DeMeo DL, Fingerlin TE, Gharib SA, Gulsvik A, Hall IP, *et al.* Genetic landscape of chronic obstructive pulmonary disease identifies heterogeneous cell-type and phenotype associations. *Nature Genetics* 2019;51:494–505.
17. Subramanian A, Narayan R, Corsello SM, Peck DD, Natoli TE, Lu X, Gould J, Davis JF, Tubelli AA, Asiedu JK, Lahr DL, Hirschman JE, Liu Z, Donahue M, Julian B, Khan M, Wadden D, Smith IC, Lam D, Liberzon A, Toder C, Bagul M, Orzechowski M, Enache OM, Piccioni F, Johnson SA, Lyons NJ, Berger AH, Shamji AF, *et al.* A Next Generation Connectivity Map: L1000 Platform and the First 1,000,000 Profiles. *Cell* 2017;171:1437-1452.e17.
