# Supplementary material for: RNA-sequencing across three matched tissues reveals shared and tissue-specific gene expression and pathway signatures of COPD
Source: Respir Res. 2019 Apr 2;20:65. doi: 10.1186/s12931-019-1032-z (PMC6446359; doi:10.1186/s12931-019-1032-z)
Supplement: Supplementary file 3 — Supplemental Tables: Additional tables to support the findings of this study. Table S4. Differential gene expression results in bronchial epithelium. Table S5. Differential gene expression results in alveolar macrophages. Table S6. Differential gene expression results in peripheral blood. (ZIP 674 kb) [file 12931_2019_1032_MOESM3_ESM.zip › supplemental_tables.pdf]

RNA-sequencing across three matched tissues reveals shared and tissue-specific gene expression and pathway signatures of COPD

Jarrett D. Morrow[1], Robert P. Chase[1], Margaret M. Parker[1], Kimberly Glass[1], Minseok Seo[1], Miguel Divo[2], Caroline A. Owen[2], Peter Castaldi[1], Dawn L. DeMeo[1,2], Edwin K. Silverman[1,2], Craig P. Hersh[1,2]

1. Channing Division of Network Medicine, Brigham and Women's Hospital, Boston, MA, 02115

2. Division of Pulmonary and Critical Care Medicine, Brigham and Women's Hospital, Boston, MA, 02115

Table S1. Demographics of study subjects

Demographics	COPD cases n=6	Controls n=11	Never-smoker controls n=4
Tissue samples:<br>large airway<br>alveolar macrophage<br>peripheral blood	 6<br>6<br>6	 11<br>11<br>11	 4<br>4<br>4
Age at enrollment (years)	63.0 (± 3.4)	64.2 (± 6.4)	56.1 (± 9.4)
Gender (female / male)	3 / 3	8 / 3	0 / 4
Race:<br>African American<br>White	 0<br>6	 6<br>5	 0<br>4
Cigarette smoking status:<br>Never Smokers<br>Former Smokers<br>Current Smokers	 0<br>2<br>4	 0<br>8<br>3	 4<br>0<br>0
Smoking History (pack-years)	36.3 (± 10.1)	26.9 (± 15.0)	NA
FEV1 % predicted	78.7 (± 19.6)	107.0 (± 13.5)	98.2 (±4.9)
FEV1/FVC	0.59 (± 0.1)	0.79 (± 0.05)	0.8 (±0.03)
Body Mass Index	24.6 (±1.4)	31.4 (±6.5)	28.7 (±3.6)
% emphysema (6 cases, 10 controls, 4 never-smokers)	7.9% (±7.1%)	1.9% (±2.3%)	0.7 (±0.4)
15th percentile of lung density histogram (6 cases, 10 controls, 4 never-smokers)	-929.5 (±23.7)	-910.2 (±10.1)	-904 (±17.8)
Adjusted lung density, sponge model adjustment (6 cases, 10 controls, 1 never-smoker)	66.3 (±16.7)	84.6±13.1	62.4 (±NA)
SRWA-Pi10 (6 cases, 9 controls, 3 never-smokers)	2.24 (±0.41)	1.79 (±0.45)	2.17 (±0.62)
Airway Wall Thickness, segmental bronchi (6 cases, 9 controls, 3 never-smokers)	1.12 (±0.19)	0.88 (±0.15)	0.96 (±0.22)
Wall Area Percent, segmental bronchi (6 cases, 9 controls, 3 never-smokers)	51.8 (±6.2)	42.6 (±7.4)	48.2 (±9.9)

Abbreviations: FEV1=forced expiratory volume in 1 sec; FVC= forced vital capacity; CT=computed tomography; SRWA-Pi10=square root wall area of a hypothetical airway with 10mm internal perimeter; Hu=Hounsfield unit

statistical tests: Fisher's Exact Test for categorical variables and Welch Two Sample t-test for continuous variables

Table S2. Number of genes with DESeq2 results overlapping across the four combined phenotype categories and three tissues (labels: phenotype category - tissue)

	**Emph blood**	**Lung function blood**	**Smoking blood**	**Airway blood**	**Emph bronch**	**Lung function bronch**	**Smoking bronch**	**Airway bronch**	**Emph macro**	**Lung function macro**	**Smoking macro**	**Airway macro**
**Emphysema - blood**	20078											
**Lung function - blood**	14691	16215										
**Smoking - blood**	13220	12491	13890									
**Airway - blood**	15009	12540	12193	15045								
**Emphysema - bronchial**	16579	16215	13129	13336	20140							
**Lung function - bronchial**	16804	14832	13890	13959	16779	19620						
**Smoking - bronchial**	20078	15433	13579	15045	18268	18119	23819					
**Airway - bronchial**	16579	16215	13129	13336	20140	16779	18268	20140				
**Emphysema - macrophage**	16804	14832	13890	13959	16779	19620	18119	16779	19620			
**Lung function - macrophage**	20064	15409	13559	15036	18200	18055	23493	18200	18055	23580		
**Smoking - macrophage**	16532	16174	13094	13295	19896	16750	18229	19896	16750	18159	20076	
**Airway - macrophage**	16773	14786	13821	13942	16750	19342	18102	16750	19342	18041	16722	19646

Table S3. Number of significant results from the DESeq2 analysis for each model and tissue (phenotype variables are color-coded to match the row labels in Figure 5; blue = lung function, red = smoking; green = emphysema, brown = airway)

**Phenotype variable**	**Phenotype description**	**Tissue**	**Number of significant results (q < 0.1)**
COPD	COPD case-control	bronch	19
COPD	COPD case-control	blood	13
COPD	COPD case-control	macro	99
FEV1pp	forced expiratory volume in 1 second - % predicted	bronch	23
FEV1pp	forced expiratory volume in 1 second - % predicted	blood	0
FEV1pp	forced expiratory volume in 1 second - % predicted	macro	0
FEV1/FVC	Ratio of forced expiratory volume in 1 sec to forced vital capacity	bronch	3
FEV1/FVC	Ratio of forced expiratory volume in 1 sec to forced vital capacity	blood	0
FEV1/FVC	Ratio of forced expiratory volume in 1 sec to forced vital capacity	macro	7
Pack-years	Pack-years of smoking	bronch	18
Pack-years	Pack-years of smoking	blood	7
Pack-years	Pack-years of smoking	macro	4
Smoking	0 non-smoker, 1 former smoker, 2 current smoker	bronch	317
Smoking	0 non-smoker, 1 former smoker, 2 current smoker	blood	1
Smoking	0 non-smoker, 1 former smoker, 2 current smoker	macro	1886
%Emph	percent emphysema	bronch	16
%Emph	percent emphysema	blood	1430
%Emph	percent emphysema	macro	4
Perc15	15th percentile of lung density histogram + 1000 Hu	bronch	0
Perc15	15th percentile of lung density histogram + 1000 Hu	blood	1692
Perc15	15th percentile of lung density histogram + 1000 Hu	macro	2
Adjusted_density	adjusted lung density, sponge model adjustment	bronch	5
Adjusted_density	adjusted lung density, sponge model adjustment	blood	263
Adjusted_density	adjusted lung density, sponge model adjustment	macro	0
Pi10 = SRWA-Pi10	square root wall area of a hypothetical airway with 10mm internal perimeter	bronch	62
Pi10 = SRWA-Pi10	square root wall area of a hypothetical airway with 10mm internal perimeter	blood	1
Pi10 = SRWA-Pi10	square root wall area of a hypothetical airway with 10mm internal perimeter	macro	12
AWT	airway wall thickness, segmental bronchi	bronch	257
AWT	airway wall thickness, segmental bronchi	blood	0
AWT	airway wall thickness, segmental bronchi	macro	388
WallAreaPct	wall area percent, segmental bronchi	bronch	159
WallAreaPct	wall area percent, segmental bronchi	blood	0
WallAreaPct	wall area percent, segmental bronchi	macro	156

Table S4. DESeq2 differential expression results for 11 phenotypes (from Table S1 in Supplemental Tables) in bronchial epithelium (see Table_S4.xls)

Table S5. DESeq2 differential expression results for 11 phenotypes (from Table S1 in Supplemental Tables) in alveolar macrophages (see Table_S5.xls)

Table S6. DESeq2 differential expression results for 11 phenotypes (from Table S1 in Supplemental Tables) in peripheral blood (see Table_S6.xls)

Table S7. Gene symbols at the intersection of results across tissue (results label: phenotype category_tissue)

**Overlapping differential gene expression results**	**Intersecting gene symbols**
emphysema_blood & emphysema_bronch	*FCN1*
emphysema_blood & emphysema_macro	*NBPF3*
smoking_blood & smoking_macro	*IFIT3, IFIT2, RSAD2, FZD6*
smoking_bronch & smoking_macro	*CCDC81, VGLL3, CYP1B1, CCL24, KIF21A, CYP1B1-AS1, UCHL1, CRISP3, CNTN5, DTNA, LTBP1, SLIT2, PROM1, AHRR, NECTIN3, SCGB1A1, PID1, LTF, AC112198.2, UPK1B, CNTD1, PROK2, SERTAD4, PCDH17, SAA1, ABCA13, CCL20, LINC02300, MAK, SYNM, SPAG1, C1orf158, C1orf116, CCDC181, MNS1, BIRC3, MAP3K19, TSPAN2, TMC5, OSBPL6, LUCAT1, ZNF860, SERPINB10, SOD2, CLDN16, TMPRSS4, SERPINB4, FBXW10, MYLK3, ZNF204P, IL5RA, FAM3B, CERKL, FAM216B, GBP1, DPYSL3, AKR1C2*
lungfunc_blood & lungfunc_macro	*LINC02384, HLA-V*
airway_bronch & airway_macro	*MIR3142HG, ABLIM1, SNORA49, SLC7A11, CYP3A5, PRSS12, ABCA13, C1orf87, AGR2, SCGB1A1, MPDZ, SCARNA13, CKAP4, CLEC4F, TRBC1, SPOCK2, SERPINB5*

Table S8. Results from cross-tissue gene and pathway enrichment for results from each phenotype category

	Genes			Pathways		
	**Epithelium--Blood**	**Epithelium--Macrophage**	**Blood--Macrophage**	**Epithelium--Blood**	**Epithelium--Macrophage**	**Blood--Macrophage**
**Lung Function**	1	1	0.003	0.009	< 0.0001	1
**Emphysema**	0.7	1	0.4	< 0.0001	< 0.0001	0.4
**Airway disease**	1	0.002	1	0.006	< 0.0001	1
**Smoking**	1	< 0.0001	0.001	< 0.0001	< 0.0001	0.003

p-values from hypergeometric tests of enrichment

Table S9. Gene set enrichment analysis pathways at the intersections across tissue (set labels: phenotype category - tissue)

**Intersecting sets**	**Database**	**Pathway**
emphysema-blood<br>& emphysema-bronch<br>& emphysema-macro	REACTOME<br>KEGG<br>KEGG	HEMOSTASIS,<br>CHEMOKINE_SIGNALING_PATHWAY,<br>CYTOKINE_CYTOKINE_RECEPTOR_INTERACTION
emphysema-bronch<br>& emphysema-macro	REACTOME<br>REACTOME<br>REACTOME<br>REACTOME<br>REACTOME<br>REACTOME<br>REACTOME<br>REACTOME<br>REACTOME<br>REACTOME<br>REACTOME<br>REACTOME<br>REACTOME<br>REACTOME<br>REACTOME<br>REACTOME<br>REACTOME<br>REACTOME	CELL_CYCLE,<br>CELL_CYCLE_MITOTIC,<br>DNA_REPLICATION,<br>MITOTIC_M_M_G1_PHASES,<br>ADAPTIVE_IMMUNE_SYSTEM,<br>G1_S_TRANSITION,<br>MITOTIC_G1_G1_S_PHASES,<br>CELL_CYCLE_CHECKPOINTS,<br>CLASS_I_MHC_MEDIATED_ANTIGEN_PROCESSING_PRESENTATION,<br>SYNTHESIS_OF_DNA,<br>M_G1_TRANSITION,<br>MITOTIC_PROMETAPHASE,<br>ANTIGEN_PROCESSING_CROSS_PRESENTATION,<br>S_PHASE,<br>CYTOKINE_SIGNALING_IN_IMMUNE_SYSTEM,<br>ORC1_REMOVAL_FROM_CHROMATIN,<br>ASSEMBLY_OF_THE_PRE_REPLICATIVE_COMPLEX,<br>CHEMOKINE_RECEPTORS_BIND_CHEMOKINES
emphysema-blood<br>& emphysema-bronch	REACTOME<br>REACTOME<br>REACTOME<br>REACTOME<br>REACTOME<br>KEGG<br>REACTOME<br>KEGG<br>KEGG<br>KEGG<br>REACTOME<br>REACTOME<br>KEGG<br>KEGG<br>REACTOME<br>REACTOME<br>KEGG<br>KEGG<br>REACTOME<br>KEGG<br>REACTOME<br>KEGG<br>REACTOME<br>KEGG<br>REACTOME<br>REACTOME<br>REACTOME<br>KEGG<br>KEGG<br>KEGG<br>REACTOME<br>REACTOME<br>REACTOME<br>REACTOME<br>REACTOME<br>REACTOME<br>REACTOME<br>REACTOME<br>REACTOME<br>KEGG	PLATELET_ACTIVATION_SIGNALING_AND_AGGREGATION,<br>RNA_POL_I_PROMOTER_OPENING,<br>GPCR_DOWNSTREAM_SIGNALING,<br>SIGNALING_BY_GPCR,<br>AMYLOIDS,<br>ENDOCYTOSIS,<br>RESPONSE_TO_ELEVATED_PLATELET_CYTOSOLIC_CA2_,<br>REGULATION_OF_ACTIN_CYTOSKELETON,<br>MAPK_SIGNALING_PATHWAY,<br>SYSTEMIC_LUPUS_ERYTHEMATOSUS,<br>DEVELOPMENTAL_BIOLOGY,<br>AXON_GUIDANCE,<br>FC_GAMMA_R_MEDIATED_PHAGOCYTOSIS,<br>LYSOSOME,<br>SIGNALLING_BY_NGF,<br>G_ALPHA_I_SIGNALLING_EVENTS,<br>LEUKOCYTE_TRANSENDOTHELIAL_MIGRATION,<br>AXON_GUIDANCE,<br>INTEGRIN_CELL_SURFACE_INTERACTIONS,<br>FOCAL_ADHESION,<br>PACKAGING_OF_TELOMERE_ENDS,<br>PATHWAYS_IN_CANCER,<br>METABOLISM_OF_CARBOHYDRATES,<br>NEUROTROPHIN_SIGNALING_PATHWAY,<br>SIGNAL_TRANSDUCTION_BY_L1,<br>SIGNALING_BY_SCF_KIT,<br>PHOSPHOLIPID_METABOLISM,<br>FC_EPSILON_RI_SIGNALING_PATHWAY,<br>LEISHMANIA_INFECTION,<br>ACUTE_MYELOID_LEUKEMIA,<br>SEMAPHORIN_INTERACTIONS,<br>MEIOTIC_SYNAPSIS,<br>SIGNALING_BY_ILS,<br>METABOLISM_OF_LIPIDS_AND_LIPOPROTEINS,<br>CLASS_A1_RHODOPSIN_LIKE_RECEPTORS,<br>ACTIVATION_OF_CHAPERONE_GENES_BY_XBP1S,<br>PEPTIDE_LIGAND_BINDING_RECEPTORS,<br>MEIOTIC_RECOMBINATION,<br>L1CAM_INTERACTIONS,<br>VEGF_SIGNALING_PATHWAY,

	REACTOME<br>REACTOME<br>KEGG<br>KEGG<br>KEGG<br>REACTOME<br>KEGG<br>KEGG<br>REACTOME<br>REACTOME<br>REACTOME<br>REACTOME<br>KEGG<br>REACTOME<br>REACTOME<br>REACTOME<br>REACTOME<br>REACTOME<br>REACTOME<br>KEGG<br>KEGG<br>REACTOME<br>REACTOME<br>REACTOME<br>REACTOME<br>REACTOME<br><br>KEGG<br>KEGG<br>KEGG<br>KEGG<br>KEGG<br>REACTOME<br>KEGG<br>KEGG<br>KEGG<br>KEGG<br>KEGG<br>KEGG<br>REACTOME<br>KEGG<br>REACTOME<br>KEGG	NGF_SIGNALLING_VIA_TRKA_FROM_THE_PLASMA_MEMBRANE,<br>SIGNALING_BY_RHO_GTPASES,<br>PHOSPHATIDYLINOSITOL_SIGNALING_SYSTEM,<br>INSULIN_SIGNALING_PATHWAY,<br>FRUCTOSE_AND_MANNOSE_METABOLISM,<br>GLUCOSE_METABOLISM,<br>NATURAL_KILLER_CELL_MEDIATED_CYTOTOXICITY,<br>ADIPOCYTOKINE_SIGNALING_PATHWAY,<br>GPCR_LIGAND_BINDING,<br>GENERIC_TRANSCRIPTION_PATHWAY,<br>TRANSLATION,<br>SRP_DEPENDENT_COTRANSLATIONAL_PROTEIN_TARGETING_TO_MEMBRANE,<br>RIBOSOME,<br>3_UTR_MEDIATED_TRANSLATIONAL_REGULATION,<br>PEPTIDE_CHAIN_ELONGATION,<br>INFLUENZA_VIRAL_RNA_TRANSCRIPTION_AND_REPLICATION,<br>METABOLISM_OF_RNA,<br>INFLUENZA_LIFE_CYCLE,<br>NONSENSE_MEDIATED_DECAY_ENHANCED_BY_THE_EXON_JUNCTION_COMPLEX,<br>TOLL_LIKE_RECEPTOR_SIGNALING_PATHWAY,<br>EPITHELIAL_CELL_SIGNALING_IN_HELICOBACTER_PYLORI_INFECTION,<br>GASTRIN_CREB_SIGNALLING_PATHWAY_VIA_PKC_AND_MAPK,<br>FORMATION_OF_THE_TERNARY_COMPLEX_AND_SUBSEQUENTLY_THE_43S_COMPLEX,<br>METABOLISM_OF_MRNA,<br>METABOLISM_OF_PROTEINS,<br>ACTIVATION_OF_THE_MRNA_UPON_BINDING_OF_THE_CAP_BINDING_COMPLEX_AND_EIFS_AND_SU<br>BSEQUENT_BINDING_TO_43S,<br>ERBB_SIGNALING_PATHWAY,<br>PANCREATIC_CANCER,<br>T_CELL_RECEPTOR_SIGNALING_PATHWAY,<br>CHRONIC_MYELOID_LEUKEMIA,<br>ARRHYTHMOGENIC_RIGHT_VENTRICULAR_CARDIOMYOPATHY_ARVC,<br>LATENT_INFECTION_OF_HOMO_SAPIENS_WITH_MYCOBACTERIUM_TUBERCULOSIS,<br>NON_SMALL_CELL_LUNG_CANCER,<br>GALACTOSE_METABOLISM,<br>DILATED_CARDIOMYOPATHY,<br>HEMATOPOIETIC_CELL_LINEAGE,<br>GNRH_SIGNALING_PATHWAY,<br>ADHERENS_JUNCTION,<br>SIGNALING_BY_EGFR_IN_CANCER,<br>LONG_TERM_DEPRESSION,<br>SIGNALING_BY_NOTCH,<br>GLYCOLYSIS_GLUCONEOGENESIS
smoking-blood<br>& smoking-macro<br>& smoking-bronch	REACTOME<br>REACTOME<br>REACTOME<br>REACTOME	RNA_POL_I_PROMOTER_OPENING,<br>AMYLOIDS,<br>PACKAGING_OF_TELOMERE_ENDS,<br>RNA_POL_I_TRANSCRIPTION
smoking-blood<br>& smoking-macro	REACTOME	ADAPTIVE_IMMUNE_SYSTEM
smoking-blood<br>& smoking-bronch	KEGG	SYSTEMIC_LUPUS_ERYTHEMATOSUS
smoking-bronch<br>& smoking-macro	REACTOME<br>REACTOME<br>REACTOME<br>REACTOME<br>KEGG<br>REACTOME<br>REACTOME<br>REACTOME<br>REACTOME<br>REACTOME<br>REACTOME<br>REACTOME<br>REACTOME<br>KEGG<br>REACTOME<br>REACTOME	METABOLISM_OF_PROTEINS,<br>PEPTIDE_CHAIN_ELONGATION,<br>INFLUENZA_VIRAL_RNA_TRANSCRIPTION_AND_REPLICATION,<br>3_UTR_MEDIATED_TRANSLATIONAL_REGULATION,<br>RIBOSOME,<br>TRANSLATION,<br>SRP_DEPENDENT_COTRANSLATIONAL_PROTEIN_TARGETING_TO_MEMBRANE,<br>NONSENSE_MEDIATED_DECAY_ENHANCED_BY_THE_EXON_JUNCTION_COMPLEX,<br>MEIOTIC_RECOMBINATION,<br>INFLUENZA_LIFE_CYCLE,<br>TELOMERE_MAINTENANCE,<br>RNA_POL_I_RNA_POL_III_AND_MITOCHONDRIAL_TRANSCRIPTION,<br>MEIOSIS,<br>LYSOSOME,<br>METABOLISM_OF_MRNA,<br>FORMATION_OF_THE_TERNARY_COMPLEX_AND_SUBSEQUENTLY_THE_43S_COMPLEX,

	REACTOME<br>REACTOME	ACTIVATION_OF_THE_MRNA_UPON_BINDING_OF_THE_CAP_BINDING_COMPLEX_AND_EIFS_AND_SUBSEQUENT_BINDING_TO_43S,<br>DEPOSITION_OF_NEW_CENPA_CONTAINING_NUCLEOSOMES_AT_THE_CENTROMERE
lungfunc-blood & lungfunc-bronch	REACTOME<br>REACTOME<br>REACTOME<br>KEGG<br>REACTOME<br>KEGG	HEMOSTASIS,<br>METABOLISM_OF_LIPIDS_AND_LIPOPROTEINS,<br>SIGNALING_BY_ILS,<br>INSULIN_SIGNALING_PATHWAY,<br>ADAPTIVE_IMMUNE_SYSTEM,<br>NEUROTROPHIN_SIGNALING_PATHWAY
lungfunc-macro & lungfunc-bronch	REACTOME<br>REACTOME<br>REACTOME<br>REACTOME<br>KEGG<br>REACTOME<br>REACTOME<br>KEGG<br>REACTOME<br>REACTOME<br>KEGG<br>REACTOME<br>KEGG<br>REACTOME<br>REACTOME<br>REACTOME<br>REACTOME<br>REACTOME<br>REACTOME<br>REACTOME<br>KEGG<br>REACTOME<br>REACTOME<br>REACTOME<br>REACTOME<br>REACTOME<br>REACTOME<br>REACTOME<br>REACTOME<br>REACTOME	METABOLISM_OF_PROTEINS,<br>TRANSLATION,<br>METABOLISM_OF_RNA,<br>SRP_DEPENDENT_COTRANSLATIONAL_PROTEIN_TARGETING_TO_MEMBRANE,<br>RIBOSOME,<br>METABOLISM_OF_MRNA,<br>INFLUENZA_LIFE_CYCLE,<br>ALZHEIMERS_DISEASE,<br>3_UTR_MEDIATED_TRANSLATIONAL_REGULATION,<br>TCA_CYCLE_AND_RESPIRATORY_ELECTRON_TRANSPORT,<br>OXIDATIVE_PHOSPHORYLATION,<br>INFLUENZA_VIRAL_RNA_TRANSCRIPTION_AND_REPLICATION,<br>HUNTINGTONS_DISEASE,<br>CLASS_I_MHC_MEDIATED_ANTIGEN_PROCESSING_PRESENTATION,<br>METABOLISM_OF_AMINO_ACIDS_AND_DERIVATIVES,<br>NONSENSE_MEDIATED_DECAY_ENHANCED_BY_THE_EXON_JUNCTION_COMPLEX,<br>PEPTIDE_CHAIN_ELONGATION,<br>RESPIRATORY_ELECTRON_TRANSPORT_ATP_SYNTHESIS_BY_CHEMIOSMOTIC_COUPLING_AND_HEAT_PRODUCTION_BY_UNCOUPLING_PROTEINS_,<br>ANTIGEN_PROCESSING_UBIQUITINATION_PROTEASOME_DEGRADATION,<br>ACTIVATION_OF_THE_MRNA_UPON_BINDING_OF_THE_CAP_BINDING_COMPLEX_AND_EIFS_AND_SUBSEQUENT_BINDING_TO_43S,<br>PARKINSONS_DISEASE,<br>FORMATION_OF_THE_TERNARY_COMPLEX_AND_SUBSEQUENTLY_THE_43S_COMPLEX,<br>HIV_INFECTION,<br>RESPIRATORY_ELECTRON_TRANSPORT,<br>SIGNALING_BY_WNT,<br>HIV_LIFE_CYCLE,<br>REGULATION_OF_MRNA_STABILITY_BY_PROTEINS_THAT_BIND_AU_RICH_ELEMENTS,<br>METABOLISM_OF_NON_CODING_RNA,<br>PROCESSING_OF_CAPPED_INTRON_CONTAINING_PRE_MRNA,<br>HOST_INTERACTIONS_OF_HIV_FACTORS
airway-blood & airway-bronch	REACTOME<br>REACTOME<br>REACTOME<br>REACTOME	CYTOKINE_SIGNALING_IN_IMMUNE_SYSTEM,<br>INTERFERON_ALPHA_BETA_SIGNALING,<br>INTERFERON_SIGNALING,<br>ADAPTIVE_IMMUNE_SYSTEM
airway-macro & airway-bronch	REACTOME<br>REACTOME<br>REACTOME<br>REACTOME<br>REACTOME<br>REACTOME<br>REACTOME<br>REACTOME<br>REACTOME<br>REACTOME<br>REACTOME<br>KEGG<br>KEGG<br>KEGG<br>REACTOME<br>REACTOME<br>REACTOME<br>REACTOME<br>KEGG<br>REACTOME<br>REACTOME<br>KEGG	METABOLISM_OF_PROTEINS,<br>CELL_CYCLE,<br>CELL_CYCLE_MITOTIC,<br>TRANSLATION,<br>METABOLISM_OF_RNA,<br>METABOLISM_OF_MRNA,<br>DNA_REPLICATION,<br>SRP_DEPENDENT_COTRANSLATIONAL_PROTEIN_TARGETING_TO_MEMBRANE,<br>MITOTIC_M_M_G1_PHASES,<br>INFLUENZA_LIFE_CYCLE,<br>TRANSCRIPTION,<br>HUNTINGTONS_DISEASE,<br>LYSOSOME,<br>ALZHEIMERS_DISEASE,<br>3_UTR_MEDIATED_TRANSLATIONAL_REGULATION,<br>MITOTIC_G1_G1_S_PHASES,<br>INFLUENZA_VIRAL_RNA_TRANSCRIPTION_AND_REPLICATION,<br>TCA_CYCLE_AND_RESPIRATORY_ELECTRON_TRANSPORT,<br>RIBOSOME,<br>NONSENSE_MEDIATED_DECAY_ENHANCED_BY_THE_EXON_JUNCTION_COMPLEX,<br>CLASS_I_MHC_MEDIATED_ANTIGEN_PROCESSING_PRESENTATION,<br>CELL_CYCLE,

	REACTOME	G1_S_TRANSITION,
	KEGG	OXIDATIVE_PHOSPHORYLATION,
	REACTOME	PEPTIDE_CHAIN_ELONGATION,
	REACTOME	CELL_CYCLE_CHECKPOINTS, REACTOME_METABOLISM_OF_AMINO_ACIDS_AND_DERIVATIVES,
	KEGG	PARKINSONS_DISEASE,
	REACTOME	RESPIRATORY_ELECTRON_TRANSPORT_ATP_SYNTHESIS_BY_CHEMIOSMOTIC_COUPLING_AND_HEAT_PRODUCTION_BY_UNCOUPLING_PROTEINS_,
	REACTOME	UNFOLDED_PROTEIN_RESPONSE,
	REACTOME	S_PHASE,
	REACTOME	M_G1_TRANSITION,
	REACTOME	SYNTHESIS_OF_DNA,
	REACTOME	HIV_INFECTION,
	REACTOME	SIGNALING_BY_THE_B_CELL_RECEPTOR_BCR,
	REACTOME	ACTIVATION_OF_THE_MRNA_UPON_BINDING_OF_THE_CAP_BINDING_COMPLEX_AND_EIFS_AND_SUBSEQUENT_BINDING_TO_43S,
	REACTOME	REGULATION_OF_MRNA_STABILITY_BY_PROTEINS_THAT_BIND_AU_RICH_ELEMENTS,
	REACTOME	DOWNSTREAM_SIGNALING_EVENTS_OF_B_CELL_RECEPTOR_BCR,
	REACTOME	RESPIRATORY_ELECTRON_TRANSPORT,
	REACTOME	ANTIGEN_PROCESSING_UBIQUITINATION_PROTEASOME_DEGRADATION,
	REACTOME	MITOTIC_PROMETAPHASE,
	REACTOME	ASSEMBLY_OF_THE_PRE_REPLICATIVE_COMPLEX,
	REACTOME	ANTIGEN_PROCESSING_CROSS_PRESENTATION,
	REACTOME	FORMATION_OF_THE_TERNARY_COMPLEX_AND_SUBSEQUENTLY_THE_43S_COMPLEX,
	REACTOME	P53_DEPENDENT_G1_DNA_DAMAGE_RESPONSE,
	REACTOME	ORC1_REMOVAL_FROM_CHROMATIN,
	REACTOME	DESTABILIZATION_OF_MRNA_BY_AUF1_HNRNP_D0,
	REACTOME	ER_PHAGOSOME_PATHWAY,
	REACTOME	APC_C_CDC20_MEDIATED_DEGRADATION_OF_MITOTIC_PROTEINS,
	REACTOME	REGULATION_OF_MITOTIC_CELL_CYCLE,
	REACTOME	SIGNALING_BY_WNT,
	REACTOME	APC_C_CDH1_MEDIATED_DEGRADATION_OF_CDC20_AND_OTHER_APC_C_CDH1_TARGETED_PROTEINS_IN_LATE_MITOSIS_EARLY_G1,
	REACTOME	P53_INDEPENDENT_G1_S_DNA_DAMAGE_CHECKPOINT,
	REACTOME	CDT1_ASSOCIATION_WITH_THE_CDC6_ORC_ORIGIN_COMPLEX, REACTOME_HIV_LIFE_CYCLE,
	REACTOME	CYCLIN_E_ASSOCIATED_EVENTS_DURING_G1_S_TRANSITION_,
	REACTOME	CDK_MEDIATED_PHOSPHORYLATION_AND_REMOVAL_OF_CDC6,
	REACTOME	PROCESSING_OF_CAPPED_INTRON_CONTAINING_PRE_MRNA,
	REACTOME	CROSS_PRESENTATION_OF_SOLUBLE_EXOGENOUS_ANTIGENS_ENDOSOMES,
	REACTOME	AUTODEGRADATION_OF_THE_E3_UBIQUITIN_LIGASE_COP1,
	KEGG	UBIQUITIN_MEDIATED_PROTEOLYSIS, REACTOME_SCFSKP2_MEDIATED_DEGRADATION_OF_P27_P21,
	REACTOME	REGULATION_OF_ORNITHINE_DECARBOXYLASE_ODC,
	REACTOME	SCF_BETA_TRCP_MEDIATED_DEGRADATION_OF_EMI1,
	REACTOME	LATE_PHASE_OF_HIV_LIFE_CYCLE,
	REACTOME	MRNA_SPLICING,
	REACTOME	MRNA_PROCESSING,
	REACTOME	AUTODEGRADATION_OF_CDH1_BY_CDH1_APC_C,
	REACTOME	VIF_MEDIATED_DEGRADATION_OF_APOBEC3G,
	REACTOME	G2_M_CHECKPOINTS,
	REACTOME	E2F_MEDIATED_REGULATION_OF_DNA_REPLICATION,
	KEGG	FC_GAMMA_R_MEDIATED_PHAGOCYTOSIS,
	KEGG	SPLICEOSOME,
	REACTOME	PROTEIN_FOLDING,
	REACTOME	DNA_REPAIR,
	REACTOME	RNA_POL_II_TRANSCRIPTION,
	REACTOME	MITOCHONDRIAL_PROTEIN_IMPORT,
	REACTOME	MRNA_SPLICING_MINOR_PATHWAY,
	REACTOME	PREFOLDIN_MEDIATED_TRANSFER_OF_SUBSTRATE_TO_CCT_TRIC,
	REACTOME	TRNA_AMINOACYLATION

Table S10. Overlapping genes from significant (q-value < 0.1) DESeq2 differential gene expression results and the findings from previous studies (rows labeled by: phenotype variable - tissue)

	**Lung DGE up** (1)	**Lung DGE down** (1)	**B cell** (1)	**Lung emph up** (1)	**Lung emph down** (1)	**Lung EWAS** (2)	**GWAS** (3)
**COPD - bronch**			CD28				ADAMTSL3
**COPD - blood**						PTPRE	IER3
**COPD - macro**	FCRL2, FCGR2B	PI4K2B, EIF3F	STAP1, FCRL2	FCGR2B	PI4K2B, EIF3F, CD101	KALRN, ACSM1	
**FEV1pp - bronch**		EXOC8			EXOC8		
**Pack-years - macro**	PLA2G7						
**Smoking - bronch**	SV2B	CIT, RASGEF1B			FXR1, FTH1	THSD7A, DTNA, SLC7A8, BOK, OSBPL6, FERMT1	TIMP3, PID1, AZGP1, CNGB1
**Smoking - macro**	POU2AF1, PLA2G7, FCGR2B, CD24, FCRL2, SERPINE2	CLEC2D, MYO5B, KRT8, LY75, FBXO5	POU2AF1, FCRL2, TLR10, CD28, AIM2, LAX1, COCH, HLA-DOB, FAM46C	C1S, CD8A, SYTL2, FCGR2B, FAM20C, SULF1, SAMD3, CALD1, MAP1A, CACNA1C, GZMK, LIMA1, EMILIN1, OSBPL10, PYHIN1, CD5, PLTP	XAF1, ACAD8, SCD, CD101, LY75	DIXDC1, DNAH6, TMEM163, LRIG1, RPS6KA2, OSBPL6, PLCB4, CCDC80, CNN3, FOXP2, SHANK2, IL7, CACHD1, TEAD1, FILIP1, PLXNB1, FARP1, DTNA, NUP210L, RAB36, ALDH2, RNASE1, C5orf49, THSD4, MSI2, ERN2, NR3C2, TMEM212, SPIDR, PTPN6, TEKT2, IRF4, RASGRP1, TGFBI, AGBL4, KALRN, ZNF608, TEX14, VILL, SORT1, CCDC68, DUSP2, RUNX2, LHFPL2, SMAD3	TNS1, C1orf87, PPP1R3B, MECOM, ITGA2, CHRNA5, SLC27A2, HYKK, DYDC2, PID1, ID4, CALHM6, THSD4, DSP, RBMS3, PARVA, GSDMB, RARB, FAM49A
**%Emph - blood**	ZNF101,	METTL18,	TMEM156,	FPGT	MS4A7, PSMC6,	CLN6, ZNF136,	CCDC91,

	FPGT, EEF1B2, MS4A1, MTHFD2L	DNTTIP2, HSP90AA1, ZCCHC10, HSPA13, PSMC6, CCDC82, XRCC4, PI4K2B, CBWD1, FBXO5, SOCS4, HMGB1, ADH5, SREK1IP1, TPRKB, DNAJB14, NUDT21, SNRPG, VEZT, HNRNPA1, C11orf58, TAF9, RASGEF1B	FAM69A, STAP1, AIM2, BTLA, COCH, MS4A1, EAF2		PI4K2B, LIN7C, NPM1, SOCS4, CCDC91, HMGB1, COMMD8, EIF4E, FXR1, DNAJB14, NUDT21, ZNF354B, NCF1, PFKFB4, HNRNPA3, SRSF3, HNRNPA1, ZBTB8OS, C11orf58, TAF9, ZNRF2, TAF1D	ZNF608, PLEKHO2, TP53RK, SLC43A2, ZBTB25, ALOX5, KCNQ1, PSD4, IQSEC1, GNAI2, RBM12B	DRAM2, ITPK1, IER3, PADI2, HIKESHI, PPA2, MRPS35, PSMA4, HSPA4, PPM1K, SSR1, ATG7, VTA1, ADAM19
**Perc15 - blood**	EEF1B2, MS4A1, MTHFD2L, ZNF101	ZCCHC10, PSMC6, SNRPG, PI4K2B, HSPA13, CBWD1, METTL18, HSP90AA1, HMGB1, SREK1IP1, XRCC4, VEGFA, TAGLN2, TPRKB, NUDT21, ADH5, SOCS4, DNAJB14, C11orf58, DNTTIP2, TAF9, CCDC82, RASGEF1B, MRPL47, FBXO5, RAC1, NIPSNAP3A, C6orf62, PEX3	BTLA, STAP1, FAM69A, TMEM156, MS4A1, EAF2, RAB30		CCDC91, PSMC6, COMMD8, NCF1, MS4A7, PI4K2B, HMGB1, PFKFB4, PDE4DIP, LIN7C, EIF4E, NUDT21, SOCS4, DNAJB14, C11orf58, TAF9, ZNRF2, CLOCK, HNRNPA3, PDCD10, NPM1, XAF1, SRSF3, ZBTB8OS, MLX, ZNF354B, TAF1D, RAC1, C6orf62, FXR1, PEX3	CLN6, ALOX5, PLEKHO2, PSD4, SLC43A2, KCNQ1, ARID3A, FCGRT, LSP1, GNAI2, KIF13A, IL6R, ZBTB25, CTDSP1, PTPRE, GLT1D1, SBNO2, IQSEC1, ATP6V1B2, HACE1, SVIL, VAV1, KSR1, TRAF5, TP53RK	CCDC91, ITPK1, DRAM2, PSMA4, PPA2, HIKESHI, RRP15, RIN3, PPM1K, MRPS35, VTA1
**Adjusted_density - bronch**						DHRS7	

**Adjusted_density - blood**	ZNF101	ZCCHC10, HSP90AA1, TPRKB, DNTTIP2, PSMC6, HSPA13	STAP1		MS4A7, PSMC6, COMMD8, LIN7C		
**Pi10 - bronch**							FAM49A
**AWT - bronch**		S100A10		KLF12		FUT3, THSD7A, BOK, ZNF608, FRMD4A	PTHLH, C1orf87
**AWT - macro**	FCGR2B, CD24, PLA2G7, FCRL2	KRT8, MYO5B	FCRL2, CD28	FCGR2B, CD8A, SAMD3, SYTL2		RASGRP1, ELF5, PLCB4, IL7, CACHD1, HOXB4, KALRN, DNAH6, HOXB3, IL6R	PID1, MECOM, CYFIP2, C1orf87, ITGA2
**WallAreaPct - bronch**				ANXA6, SULF1		UGCG, FRMD4A	PTHLH
**WallAreaPct - macro**	FCRL2, EEF1B2	SNRPG	FCRL2		NPM1	RASGRP1, KLHL29, AMOTL1	CYFIP2

DGE = differential gene expression; EWAS = epigenome-wide association study; GWAS = genome-wide association study

COPD = case-control status; FEV1=forced expiratory volume in 1 sec

pack-years = pack-years of smoking; smoking = 0 non-smoker, 1 former smoker, 2 current smoker

pctEmph = % emphysema; perc15 = 15th percentile of lung density histogram + 1000 Hu

adj_density = adjusted lung density, sponge model adjustment

Pi10 = SRWA-Pi10 = square root wall area of a hypothetical airway with 10mm internal perimeter

AWT = airway wall thickness; WallAreaPct = wall area percent

Table S11. CMap connectivity A549 cell-line findings for bronchial epithelium genes up-regulated and down-regulated in airway disease (no Connectivity Map classes achieved an absolute score greater than 95)

**Score**	**Compound Name**	**Description**
-99.3	lomerizine	Calcium channel blocker
-99.24	radicicol	HSP inhibitor
-99.22	CAY-10578	Casein kinase inhibitor
-99.04	flavokavain-b	Antineoplastic
-98.95	n-(3-acetamidophenyl)-3-chlorobenzamide	Glutamate receptor antagonist
-98.92	dehydroisoandosterone	GABA receptor modulator
-98.78	methocarbamol	Muscle relaxant
-98.19	aminolevulinic-acid	Oxidizing agent
-98.1	apicidin	HDAC inhibitor
-98.01	icilin	TRPV agonist
-97.75	alprazolam	Benzodiazepine receptor agonist
-97.13	nadolol	Adrenergic receptor antagonist
-96.55	digitoxigenin	ATPase inhibitor
-96.42	BIIB021	HSP inhibitor
-96.35	MLN-4924	Nedd activating enzyme inhibitor
-96.19	oxyphenonium	Cholinergic receptor antagonist
-96.08	fluocinolone	Glucocorticoid receptor agonist
-95.62	tolcapone	Catechol O methyltransferase inhibitor
-95.27	nilutamide	Androgen receptor antagonist
-95.12	westcort	Glucocorticoid receptor agonist

Table S12. CMap connectivity HCC515 cell-line findings for bronchial epithelium genes up-regulated and down-regulated in airway disease (Connectivity Map classes with absolute score greater than 95 shown at bottom)

**Score**	**Compound Name**	**Description**
-99.69	ephedrine	Adrenergic receptor agonist
-99.49	cycloserine	Bacterial cell wall synthesis inhibitor
-98.83	norethisterone	Progesterone receptor agonist
-98.69	rimantadine	Antiviral
-98.54	amcinonide	Glucocorticoid receptor agonist
-98.54	fludrocortisone	Glucocorticoid receptor agonist
-98.53	dihydrodeoxygedunin	Growth factor receptor activator
-98.44	emetine	Protein synthesis inhibitor
-98.38	17-hydroxyprogesterone-caproate	progesterone receptor agonist
-98.33	AT-7519	CDK inhibitor
-98.31	R-59022	Diacylglycerol kinase inhibitor
-98.26	SB-590885	RAF inhibitor
-98.14	narciclasine	Coflilin signaling pathway activator
-97.87	gefitinib	EGFR inhibitor
-97.81	homoharringtonine	Protein synthesis inhibitor
-97.66	protopine	Histamine receptor antagonist
-97.57	desoximetasone	Glucocorticoid receptor agonist
-97.36	thalidomide	TNF production inhibitor
-97.23	QL-X-138	MTOR inhibitor
-97.15	triamcinolone	Glucocorticoid receptor agonist
-97.13	flunisolide	Cytochrome P450 inhibitor
-96.87	AS-703026	MEK inhibitor
-96.69	budesonide	Glucocorticoid receptor agonist
-96.67	cefixime	Bacterial cell wall synthesis inhibitor
-96.63	fluocinonide	Glucocorticoid receptor agonist
-96.57	hydrocortisone	Glucocorticoid receptor agonist
-96.44	halcinonide	Glucocorticoid receptor agonist
-96.39	NU-7441	DNA dependent protein kinase inhibitor
-96.18	5'-guanidinonaltrindole	Opioid receptor antagonist
-95.95	RHO-kinase-inhibitor-II	Rho associated kinase inhibitor
-95.91	etilefrine	Adrenergic receptor agonist
-95.75	XMD-885	Leucine rich repeat kinase inhibitor
-95.69	fluticasone	Glucocorticoid receptor agonist

-95.63	teicoplanin	Bacterial cell wall synthesis inhibitor
-95.5	alclometasone	Glucocorticoid receptor agonist
-95.48	GW-583340	EGFR inhibitor
-95.41	vinpocetine	Phosphodiesterase inhibitor
-95.33	cephaeline	Protein synthesis inhibitor
-95.19	MK-212	Serotonin receptor agonist
**Connectivity Map Class**		
-96.8	NA	Rho associated kinase inhibitor
-95.75	NA	Protein synthesis inhibitor
-95.66	NA	Homeobox Gene GOF
-95.22	NA	Glucocorticoid receptor agonist

References

1. Morrow JD, Zhou X, Lao T, Jiang Z, DeMeo DL, Cho MH, Qiu W, Cloonan S, Pinto-Plata V, Celli B, Marchetti N, Criner GJ, Bueno R, Washko GR, Glass K, Quackenbush J, Choi AMK, Silverman EK, Hersh CP. Functional interactors of three genome-wide association study genes are differentially expressed in severe chronic obstructive pulmonary disease lung tissue. *Scientific Reports* 2017;7:44232.
2. Morrow JD, Cho MH, Hersh CP, Pinto-Plata V, Celli B, Marchetti N, Criner G, Bueno R, Washko G, Glass K, Choi AMK, Quackenbush J, Silverman EK, DeMeo DL. DNA methylation profiling in human lung tissue identifies genes associated with COPD. *Epigenetics* 2016;11:730–739.
3. Sakornsakolpat P, Prokopenko D, Lamontagne M, Reeve NF, Guyatt AL, Jackson VE, Shrine N, Qiao D, Bartz TM, Kim DK, Lee MK, Latourelle JC, Li X, Morrow JD, Obeidat M, Wyss AB, Zhou X, Bakke P, Barr RG, Beaty TH, Belinsky SA, Brusselle GG, Crapo JD, Jong K de, DeMeo DL, Fingerlin TE, Gharib SA, Gulsvik A, Hall IP, *et al.* Genetic landscape of chronic obstructive pulmonary disease identifies heterogeneous cell-type and phenotype associations. *Nature Genetics* 2019;51:494–505.
